# Fruit quality evaluation of different mulberry varieties

**DOI:** 10.3389/fpls.2024.1500253

**Published:** 2025-01-10

**Authors:** Jie Tian, Haichao Wen, Bingxiang Liu, Xinyuan Tian, Yibo Wu, Jingyan Yang, Bingying Zhang, Hongjiao Li

**Affiliations:** ^1^ College of Forestry, Hebei Agricultural University, Baoding, China; ^2^ Food Science and Technology College, Hebei Agricultural University, Baoding, China; ^3^ Hebei Hongya Mountain State- Owned Forest Farm, Baoding, China

**Keywords:** mulberry, fruit quality, comprehensive evaluation, principal component analysis, gray relational analysis

## Abstract

**Introduction:**

The quality of fruits has long been a key focus for breeders, and the development of scientifically sound and reasonable methods for evaluating fruit quality is of great significance in selecting superior cultivars. The mulberry tree, as a plant resource that serves both medicinal and dietary purposes, contains rich nutritional components and various bioactive compounds. These include properties such as immune enhancement, lipid-lowering effects, and anti-tumor activities.

**Methods:**

Therefore, to select mulberry varieties with superior quality and adapt to the diversification trends in mulberry development, this study uses 21 mulberry varieties to analyze and compare differences in fruit appearance quality, nutritional quality, functional components, and antioxidant capacity. Principal Component Analysis (PCA) was employed to identify core evaluation indices, and the Entropy Weight Method was used to assign weights based on these core quality indices. Subsequently, Grey Relational Analysis (GRA) was used for a comprehensive evaluation of the fruit quality of the 21 mulberry varieties.

**Results:**

The results indicate that, in terms of appearance quality, varieties such as ‘Ri Ben Guo Sang’, ‘Hong Guo 1’, ‘Lv Shen Zi’, ‘He Lan Sang’, and ‘Ju Shen’ stand out overall. In terms of nutritional quality, ‘Tang 10’ has relatively higher levels of free amino acids and soluble proteins, but its solid-acid ratio is the lowest, which affects the taste of the fruit. Overall, varieties such as ‘Jiang Mi Guo Sang’, ‘Bai Shen 2’, ‘Ji Gui Hua’, ‘Xiao Bai E’, ‘Da Bai E’, and ‘Da Yi Bai’ stand out in terms of comprehensive quality. Regarding functional components, the four varieties—’Lv Shen Zi’, ‘Hei Zhen Zhu’, ‘He Lan Sang’, and ‘Da 10’—are prominent across all indicators. In terms of antioxidant capacity, ‘Jiang Mi Guo Sang’, ‘Hong Guo 1’, ‘Xiao Bai E’, ‘Da Bai E’, and ‘Da Yi Bai’ rank relatively high, which largely overlaps with the varieties selected for their nutritional quality. Regarding fruit enzyme activity, ‘Ri Ben Guo Sang’, ‘Hong Guo 1’, ‘Lv Shen Zi 1’, ‘Lv Shen Zi 2’, ‘He Lan Sang’, and ‘Da 10’ show high enzyme activities. Finally, based on Principal Component Analysis (PCA), the fruit’s appearance quality, nutritional quality, functional components, and antioxidant capacity were categorized into seven principal components, covering 12 indicators, with a cumulative variance contribution rate of 88.424%. The Entropy Weight Method was used to assign weights to these 12 indicators, and the final correlation degree was calculated using Grey Relational Analysis (GRA), with a range from 0.406 to 0.817.

**Conclusion:**

This study suggests that varieties such as ‘Da 10’, ‘Feng Guo Sang’, ‘He Lan Sang’, ‘Lv Shen Zi’, and ‘Ri Ben Guo Sang’ exhibit superior overall fruit quality and rich nutritional value, providing a theoretical basis for the selection, development, and utilization of future mulberry fruit varieties.

## 1 Introduction

The mulberry tree (*Morus alba* L.) is a perennial, woody plant in the Moraceae family and Morus genus. This species is a significant economic forest resource in China and is widely distributed globally (Zhong et al., 2022). The fruit of the mulberry tree, commonly known as mulberry fruit, is widely consumed and is characterized by its nectar-like juice, sweet-sour fragrance, and rich nutritional content. The Chinese Ministry of Health classifies it as one of the “dual-purpose” agricultural products for both food and medicine (Hao et al., 2022). According to Singhal et al (Singhal et al., 2010), mulberries are rich in a variety of nutritional components. The carbohydrate content ranges from 7.8% to 9%, protein from 0.5% to 1.4%, fatty acids from 0.3% to 0.5%, free acids from 1.1% to 1.8%, cellulose from 0.9% to 1.3%, ash from 0.8% to 1.0%, moisture from 85% to 88%, vitamin B1 from 7.9 to 9.0 μg/100g, vitamin B2 from 165 to 179 μg/100g, and vitamin C from 11.0 to 12.5 mg/100g.

Mulberries contain a wide range of nutritional components and offer numerous health benefits, with substantial research highlighting their ability to enhance immunity, combat tumors, prevent cancer, lower blood lipids, provide antioxidant effects, promote skin health, and neutralize free radicals (Jiang and Nie, 2015; Hu et al., 2019). Fresh mulberries are an excellent source of vitamin C and a potent natural antioxidant (Sushmitha et al., 2024). Numerous studies have shown that mulberries, blackberries, and other berries are rich in phenolic compounds, such as anthocyanins and polyphenols, which significantly promote overall health (Martins et al., 2025). The results of Li et al. (Li et al., 2017) showed that these polyphenols in mulberry have blood sugar-lowering effects. Studies have demonstrated that polysaccharides in mulberries exhibit antioxidant and anti-aging effects and can scavenge superoxide anion radicals and hydroxyl radicals (Zhang et al., 2018). Recent studies have suggested that volatile compounds in mulberries exhibit health-promoting effects, including anti-inflammatory, anticancer, and antidiabetic properties (Gu et al., 2022). Certain bioactive compounds in mulberries have garnered considerable attention from researchers due to their nutritional and therapeutic properties. These properties may reduce the risk of certain chronic diseases, offering substantial opportunities for future treatments (Bhattacharjya et al., 2021). Flavonoid compounds in mulberries are known to lower blood lipids and prevent fatty liver disease (Cai et al., 2016), while alkaloid 1-DNJ and its derivatives exhibit multiple effects, including blood sugar-lowering, antiviral, and anti-inflammatory activities (Raman et al., 2016). Consequently, mulberries are widely utilized in the food processing, pharmaceutical, and other industries.

Mulberries have high nutritional value and are well-suited for development and utilization, often being dried or processed into products such as wine, syrup, canned foods, juice, jam, and beverages (Hao et al., 2022). However, due to the large variety of mulberry cultivars, significant differences exist in taste, flavor, yield, and nutritional value, leading to inconsistent quality. This variability limits their cultivation, promotion, and industrialization. Therefore, selecting superior mulberry varieties for industrial purposes has become a key focus at this stage (Sushmitha et al., 2024). Although numerous studies have been conducted on the analysis and evaluation of mulberry quality, the evaluation indicators remain relatively independent, and the comprehensive evaluation methods are often simplistic. The lack of a theoretical basis for production and development raises questions about how to comprehensively evaluate mulberries and reconcile results from different evaluation methods. These issues warrant further in-depth research.

Modern demands for superior varieties of economic forest species focus not only on high yield and stress resistance but also on taste and quality. In summary, qualitative or single-indicator evaluation methods are increasingly inadequate to meet growing demands. Principal Component Analysis (PCA) is a widely used evaluation method that standardizes and analyzes the quality traits of fruits and leaves through dimensionality reduction techniques. The principal factor value is calculated based on the variance contribution rate of the principal components and is used as the weight. The cumulative sum of the products of the principal factor values and their corresponding initial eigenvalues is divided by the sum of the initial eigenvalues to calculate the score (Vinti et al., 2020). PCA ensures that there is no correlation between the indicators obtained through recombination, thereby eliminating the impact of inter-indicator correlations on the results. While PCA offers many advantages, it also has notable drawbacks. First, in terms of contribution rate and eigenvalue, it is crucial to ensure that both the contribution rate and eigenvalue reach a certain threshold when selecting the principal components; otherwise, the selected components will fail to capture most of the original information. Secondly, from a practical perspective, the selected principal components must have real-world relevance; otherwise, the purpose of PCA cannot be fulfilled. Abdollah et al. (Abdollah et al., 2014) applied PCA to analyze 16 fruit parameters from 23 grape varieties, and the results indicated that the grape germplasm exhibited considerable fruit diversity, with high discriminant values linked to berry size.

Therefore, this study provides a comprehensive analysis of the appearance quality, nutritional quality, functional components, and antioxidant capacity of various mulberry varieties. The objective is to offer a comprehensive evaluation of mulberry varieties suitable for cultivation in Hebei Province. Correlation Analysis (CA) and Principal Component Analysis (PCA) are employed to identify the core indicators for fruit quality assessment. The Entropy Weight Method-Grey Relational Analysis (EWM-GRA) is applied to select the most suitable mulberry varieties for local cultivation in Hebei based on the composite scores of each indicator. This study aims to offer scientific guidance for the development of mulberry-based ecological agricultural systems in Hebei, which are currently being considered for localized agricultural development.

## 2 Materials and methods

### 2.1 Experimental materials

The experimental materials comprised 21 mulberry germplasm resources collected from the mulberry germplasm nursery at Hebei Agricultural University (see **Table 1**). For each variety, three randomly selected trees were observed. The trees were three years old, maintained a planting density of 1.5 m × 3.0 m, exhibited consistent growth, followed uniform management practices, and were free from pests and diseases. Fruit samples were collected when the mulberries reached the same maturity stage, were uniform in size, and were free from rot or deterioration. Some samples were rapidly frozen in liquid nitrogen and stored at -80°C for subsequent use, while others were retained for experimental methods.

**Table 1:** List of test materials.

No.	Material name	Introduced
X1	Ri Ben Guo Sang	Zhenjiang City, Jiangsu Province
X2	Jiang Mi Guo Sang	Dongguang County, Hebei Province
X3	Tian Sang 202	Zhenjiang City, Jiangsu Province
X4	Hong Guo 1	Xianyang City, Shaanxi Province
X5	Bai Shen 2	Zhenjiang City, Jiangsu Province
X6	Lv Shen Zi	Xiajin County, Shandong Province
X7	Lv Shen Zi 1	Xiajin County, Shandong Province
X8	Lv Shen Zi 2	Xiajin County, Shandong Province
X9	Hei Zhen Zhu	Maoming City, Guangdong Province
X10	Ji Gui Hua	Cangzhou City, Hebei Province
X11	Gui Hua Mi	Qian’an City, Hebei Province
X12	Xiao Bai E	Dongguang County, Hebei Province
X13	Da Bai E	Dongguang County, Hebei Province
X14	Da Yi Bai	Li County, Hebei Province
X15	Su Bai Shen	Zhenjiang City, Jiangsu Province
X16	Feng Guo Sang	Zhenjiang City, Jiangsu Province
X17	He Lan Sang	Zhenjiang City, Jiangsu Province
X18	Ju Shen	Zhenjiang City, Jiangsu Province
X19	Da 10	Zhenjiang City, Jiangsu Province
X20	Tang 10	Guangzhou City, Guangdong Province
X21	Da Ma Ya	Linqing City, Shandong Province

### 2.2 Experimental methods

#### 2.2.1 Appearance quality of fruits

The appearance quality of the fruits was assessed by measuring fruit diameter (both horizontal and vertical), fruit shape index, color, moisture content, individual fruit weight, and yield per plant (Jiao et al., 2023). For each variety, three trees were randomly selected from each planting area. Fruits were collected from the upper, middle, and lower sections of the canopy, distributed across the east, south, west, and north directions. Thirty fruits were measured using a caliper to determine their vertical and horizontal diameters. The fruit shape index was calculated as the ratio of the vertical diameter to the horizontal diameter. Individual fruit weight was measured using an analytical balance, and moisture content was determined through the drying method.

#### 2.2.2 Nutritional quality of fruits

The content of free amino acids was determined using the ninhydrin colorimetric method (Sylwia et al., 2021). A 0.1 g sample was placed in a test tube and ground with 10 mL of distilled water. The mixture was extracted using ultrasound at 40°C for 20 minutes, followed by centrifugation at 5000 rpm for 25 minutes. Subsequently, 1 mL of the supernatant was mixed with 2.0 mL of pH 6 phosphate buffer, shaken well, and allowed to stand for 5 minutes. Next, 3 mL of ninhydrin solution was added, thoroughly mixed, and incubated for 5 minutes. The mixture was heated in a boiling water bath for 30 minutes, and its absorbance was measured at 560 nm. The amino acid content was calculated using the regression equation.

The titratable acidity was measured using the phenolphthalein indicator method (Wang et al., 2024). A 1 g sample was accurately weighed and mixed with 5 mL of distilled water. The mixture was ground until it was free of coarse fibers and subsequently transferred to a test tube. After being heated in a water bath at 70-80°C for 30 minutes, the solution was diluted to 10 mL, shaken, and filtered. Five milliliters of the filtrate were placed in a beaker, and 2 to 3 drops of phenolphthalein indicator were added. The solution was titrated with 0.1 mol/L NaOH until a light pink color persisted for 30 seconds, indicating the endpoint. The volume of NaOH used was recorded, and the titration was repeated three times to obtain an average value. The soluble solid content was measured using an Abbe refractometer (Tingley, 1944). The ratio of soluble solids to titratable acidity was calculated as the soluble solid content divided by the titratable acidity.

The soluble sugar content was determined using the anthrone colorimetric method (Reis et al., 2015). A 0.1 g sample was placed in a 10 mL centrifuge tube and mixed with 4 mL of 80% ethanol. The mixture was extracted in a water bath at 80°C for 30 minutes, cooled, and subsequently centrifuged at 10,000 rpm for 10 minutes. The supernatant was carefully transferred to a new 10 mL centrifuge tube. The residue was re-extracted with 4 mL of 80% ethanol in a water bath at 80°C for 30 minutes, cooled, and centrifuged again at 10,000 rpm for 10 minutes. The two extracts were combined and diluted to a final volume of 10 mL, which served as the test solution for soluble sugar content. A 0.1 mL aliquot of the test solution was mixed sequentially with 0.9 mL of distilled water and 5 mL of anthrone reagent, and then heated in a boiling water bath for 10 minutes. The mixture was quickly cooled, and its absorbance (optical density value) was measured at a wavelength of 620 nm using a visible light spectrophotometer.

The soluble protein content was determined using the G-250 Coomassie Brilliant Blue colorimetric method (Bradford, 1976). A fresh sample of 0.3 g was accurately weighed, mixed with 5 mL of distilled water to form a homogenate on ice, and subsequently transferred to a 10 mL centrifuge tube. The mixture was centrifuged at 8000 rpm for 10 minutes, and the supernatant was collected for analysis. To the supernatant, 0.2 mL of extract, 0.8 mL of distilled water, and 5 mL of G-250 solution were added in sequence. The mixture was thoroughly mixed and allowed to stand for 5 minutes. Absorbance was measured at 595 nm, and the protein content was determined using a standard curve.

#### 2.2.3 Functional components of the fruit

The content of photosynthetic pigments was determined using the C_2_H_5_OH extraction method (Li et al., 2020). A 0.1 g sample was ground and placed in a test tube, followed by the addition of 10 mL of 95% ethanol. The mixture was incubated in the dark for 24 hours. Absorbance was measured at wavelengths of 665, 649, and 470 nm. Carotenoids were calculated using the formula (1000A470 - 2.05Ca - 114.8Cb)/245.

The flavonoid content was determined using the AlCl_3_ colorimetric method (Masturi et al., 2019). A 0.2 g sample was accurately weighed and mixed with 70% ethanol at a sample-to-solvent ratio of 1:25. The mixture was subjected to ultrasound extraction for 40 minutes, followed by centrifugation at 7000 rpm for 10 minutes. One milliliter of the supernatant was combined with 0.4 mL of 50% sodium nitrite, shaken well, and allowed to stand for 6 minutes. Subsequently, 4 mL of 4% sodium hydroxide solution was added, and the solution was diluted to a final volume of 10 mL with 70% ethanol. The mixture was allowed to stand for 15 minutes, after which the absorbance was measured at 415 nm. The flavonoid content was calculated using a regression equation.

The total polyphenol content was determined using the Folin-Ciocalteu colorimetric method (Nezam et al., 2021). A 0.1 g sample was accurately weighed and mixed with 50% ethanol at a sample-to-solvent ratio of 1:100. The mixture was subjected to ultrasound extraction for 40 minutes, followed by centrifugation at 7000 rpm for 10 minutes. One milliliter of the supernatant was combined with 1 mL of Folin-Ciocalteu reagent, shaken well, and allowed to stand for 5 minutes. Subsequently, 4 mL of 20% Na_2_CO_3_ solution was added, and the mixture was heated in a water bath at 50°C for 1 hour. The absorbance was measured at 760 nm, and the total polyphenol content was calculated using a regression equation.

The anthocyanin content was determined using the pH differential method (Luo et al., 2019). A 0.1 g sample was accurately weighed and mixed with acidified ethanol at a sample-to-solvent ratio of 1:20. The mixture was heated in a water bath at 60°C in the dark for 2 hours, followed by centrifugation at 7000 rpm for 10 minutes to obtain the supernatant. Two 10 mL test tubes were prepared, each containing 1 mL of supernatant. In one tube, the supernatant was mixed with 9 mL of pH 1 buffer solution, while in the other tube, it was mixed with 9 mL of pH 4.5 buffer solution. After 2 hours of dark stabilization, the absorbance of each sample was measured at wavelengths of 520 nm and 700 nm.

The resveratrol content was determined using the C_2_H_5_OH extraction method (Zhu et al., 2022). A 0.2 g sample was accurately weighed and mixed with 60% ethanol at a sample-to-solvent ratio of 1:15. The mixture was heated in a water bath at 60°C in the dark for 1 hour, followed by centrifugation at 7000 rpm for 10 minutes. A 0.5 mL aliquot of the supernatant was diluted 10-fold, and the absorbance was measured at a wavelength of 305 nm. The resveratrol content was calculated using a regression equation.

The chlorogenic acid content was determined using the Fe²⁺ colorimetric method (Irena et al., 2019). A 0.2 g sample was accurately weighed and mixed with 10 mL of 50% ethanol. The mixture was subjected to ultrasound extraction for 30 minutes, followed by filtration and dilution to 15 mL with 80% ethanol. A 5 mL aliquot of the diluted extract was combined with 0.5 mL of 0.2 mol/L ferric chloride. After thorough shaking, the mixture was allowed to stand for 60 minutes to develop color. The absorbance was measured at a wavelength of 755 nm, and the chlorogenic acid content was calculated using a regression equation.

The ascorbic acid content was determined using the method described in the reference (Habib et al., 2016). A 1 g fruit pulp sample was placed in a mortar, mixed with 2 mL of 50 g/L TCA solution, and ground to a slurry under ice bath conditions. The mixture was transferred to a 10 mL centrifuge tube and diluted to a final volume of 10 mL with 50 g/L TCA solution. After mixing and extracting for 10 minutes, the mixture was centrifuged, and the supernatant was collected. One milliliter of the extract was transferred to a test tube, and 1 mL of 50 g/L TCA solution and 1 mL of absolute ethanol were added. The mixture was shaken thoroughly and then sequentially treated with 0.5 mL of 0.4% phosphoric acid-ethanol solution, 1 mL of 5 g/L BP-ethanol solution, and 0.5 mL of 0.3 g/L FeCl_3_-ethanol solution. The reaction was conducted at 30°C for 60 minutes, and the absorbance was measured at a wavelength of 534 nm.

The tannin content was determined using the Folin-Ciocalteu colorimetric method (Peraman et al., 2016). A 1 g sample was mixed with 8 mL of 80% ethanol for extraction, employing ultrasound at 60°C for 30 minutes. After extraction, 1 mL of the supernatant was transferred to a test tube, to which 1 mL of Folin-Ciocalteu reagent was added. The mixture was allowed to stand for 5 minutes, after which 2 mL of 10% Na_2_CO_3_ was added. The solution was incubated in the dark at room temperature for 1 hour. Finally, the solution was diluted to a final volume of 25 mL with distilled water, and the absorbance was measured at 760 nm.

#### 2.2.4 Antioxidant quality of the fruit

The abilities to scavenge DPPH radicals, ABTS radicals, and the ferric reducing power were measured using methods described in the reference (Fatiha et al., 2022). Initially, 0.1 g of the sample was accurately weighed using an analytical balance and mixed with 50% ethanol at a sample-to-solvent ratio of 1:50. The mixture was then subjected to ultrasound extraction for 1 hour and diluted to a final volume of 10 mL for use. To measure DPPH radical scavenging ability, 0.5 mL of the extract was mixed with 2 mL of the DPPH reaction solution, shaken thoroughly, and allowed to react in the dark for 30 minutes. Absorbance was measured at a wavelength of 517 nm. For ABTS radical scavenging ability, 0.5 mL of the extract was mixed with 2 mL of the ABTS reaction solution, shaken thoroughly, and allowed to react in the dark for 6 minutes. Absorbance was measured at a wavelength of 734 nm. To determine ferric reducing power, 0.5 mL of the extract was mixed with 1 mL of distilled water and 1.8 mL of TPTZ working solution, shaken thoroughly, and allowed to react in the dark for 10 minutes. Absorbance was measured at a wavelength of 593 nm.

Initially, 0.1 g of the sample was accurately weighed using an analytical balance and mixed with 50% ethanol at a sample-to-solvent ratio of 1:50. The mixture was then subjected to ultrasound extraction for 1 hour and diluted to a final volume of 10 mL for use. To measure DPPH radical scavenging ability, 0.5 mL of the extract was mixed with 2 mL of the DPPH reaction solution, shaken thoroughly, and allowed to react in the dark for 30 minutes. Absorbance was measured at a wavelength of 517 nm. For ABTS radical scavenging ability, 0.5 mL of the extract was mixed with 2 mL of the ABTS reaction solution, shaken thoroughly, and allowed to react in the dark for 6 minutes. Absorbance was measured at a wavelength of 734 nm. To determine ferric reducing power, 0.5 mL of the extract was mixed with 1 mL of distilled water and 1.8 mL of TPTZ working solution, shaken thoroughly, and allowed to react in the dark for 10 minutes. Absorbance was measured at a wavelength of 593 nm.

The activities of superoxide dismutase (SOD), peroxidase (POD), catalase (CAT), polyphenol oxidase (PPO), and lipoxygenase (LOX) were measured using the following methods: SOD activity was assessed by the NBT photoreduction method (Giannopolitis and Ries, 1977); POD activity was determined using the guaiacol method (Samara et al., 2019); CAT activity was evaluated by the hydrogen peroxide method (Havir and Mchale, 1987); PPO activity was analyzed using the o-diphenol method (Ross and Matthew, 2014); and LOX activity was measured according to relevant literature (Viktoria and Oyvind, 2018). A sample weighing 0.3 g was accurately measured and placed in a pre-cooled mortar at 4°C. Subsequently, 5 mL of 50 mmol/L phosphate buffer at pH 7.8, also stored at 4°C, was added. The sample was rapidly and thoroughly ground to achieve a homogeneous state and was then transferred to a pre-labeled centrifuge tube. The mixture was centrifuged at 12,000 rpm for 20 minutes at 4°C. After centrifugation, the supernatant was retained at 4°C for further use.

To assess superoxide dismutase (SOD) enzyme activity, 1.3 mL of 50 mmol/L phosphate buffer (pH 7.8), 0.3 mL of 130 mmol/L methionine solution, 0.3 mL of 750 µmol/L NBT, 0.3 mL of 1 µmol/L EDTA-Na2, and 0.3 mL of 0.2 µmol/L riboflavin solution were combined. Following mixing, 500 µL of the enzyme solution was added, and the mixture was promptly exposed to 4000 lx of daylight to initiate the photoreduction reaction, with timing recorded. Upon completion of the reaction, the mixture was covered with black paper to prevent exposure to light. In the control group, 0.5 mL of distilled water was added. Control 1 was incubated in the dark, while Control 2 underwent the same reaction as the enzyme solution under 4000 lx of daylight. The dark-incubated Control 1 served as the blank control. The absorbance of the mixtures was measured at 560 nm. One unit (U) of superoxide dismutase (SOD) activity is defined as the amount that inhibits nitroblue tetrazolium (NBT) photoreduction by one unit per minute in the reaction system.

To assess peroxidase (POD) enzyme activity, combine 2.5 mL of 25 mmol/L guaiacol solution, 110 µL of enzyme solution, 220 µL of distilled water, and 0.17 mL of 250 mmol/L hydrogen peroxide solution. In the control group, substitute the enzyme extract and distilled water mixture with an equivalent volume of distilled water. Following thorough mixing, transfer the solution to a cuvette. Measure and record the initial optical density (OD) at a wavelength of 470 nm. Subsequently, incubate the mixture at 30°C in a water bath for 5 minutes and measure the final OD. One unit (U) of peroxidase (POD) activity is defined as the change in absorbance per minute.

To assess catalase (CAT) enzyme activity, add 2.8 mL of 20 mmol/L hydrogen peroxide solution and 200 µL of crude enzyme extract. In the control group, substitute the enzyme extract with an equivalent volume of distilled water. Mix the solution rapidly and measure the absorbance at 240 nm. One unit (U) of catalase (CAT) activity is defined as the change in absorbance per minute.

To assess polyphenol oxidase (PPO) activity, add 4 mL of 50 mmol/L phosphate buffer and 1 mL of 50 mmol/L o-diphenol solution, followed by 100 µL of enzyme extract. Immediately transfer the reaction mixture to a cuvette and measure the absorbance at 420 nm. One unit of activity is defined as the increase in absorbance per minute per gram of sample.

To assess lipoxygenase (LOX) activity, add 2.7 mL of 0.1 mol/L sodium phosphate buffer and 100 µL of 0.5% linoleic acid solution. Incubate the mixture at 30°C for 10 minutes, then add 200 µL of crude enzyme solution and mix thoroughly. Calibrate the spectrophotometer using distilled water as the reference, and immediately record the absorbance at 234 nm. One unit of lipoxygenase (LOX) activity is defined as the increase in absorbance of 0.01 per minute per gram of sample.

The malondialdehyde (MDA) content was determined using the thiobarbituric acid (TBA) colorimetric method (Wang et al., 2013). Pipette 2 mL of the centrifuged supernatant (for the control, add 2 mL of distilled water), then add 2 mL of 0.6% TBA solution, mix thoroughly, and incubate the mixture in a boiling water bath for 15 minutes. Rapidly cool the mixture and measure the absorbance at wavelengths of 532, 600, and 450 nm. The calculation formula is as follows: MDA content (µmol/g fresh weight) = (6.45 × (D532 - D600) - 0.56 × D450) × V/W, where V represents the total volume of the extract (mL) and W represents the sample weight (g).

### 2.3 Data processing and analysis

Data processing and summarization were conducted using Excel 2016, while data analysis was performed with SPSS 26.0, utilizing Duncan’s new multiple range test for significance analysis. For the comprehensive evaluation, principal component analysis (PCA) was employed to identify core evaluation indicators. Based on these core quality indicators, the entropy weight method was applied to assign weights, and gray relational analysis was used to evaluate different mulberry tree varieties comprehensively.

The experimental data were normalized and utilized as comparison sequences, denoted as Xi (I = 0, 1, …, 21), representing patterns I to XXI. The optimal values of each indicator were selected as the optimal sequence X0 (K), with a distinguishing coefficient ρ = 0.5, and k = {1, 2, …, m}. The correlation coefficient SSS between each sequence and the reference sequence was calculated using Formula (1).


(1) $$S_{i}\left(k\right)=\frac{min_{i}min_{k}\left|x_{0}\left(k\right)-x_{i}\left(k\right)\right|+\rho \cdot max_{i}max_{k}\left|x_{0}\left(k\right)-x_{i}\left(k\right)\right|}{\left|x_{0}\left(k\right)-x_{i}\left(k\right)\right|+\rho \cdot max_{i}max_{k}\left|x_{0}\left(k\right)-x_{i}\left(k\right)\right|}$$

The entropy weight method was used to determine the weights W of each indicator. The weighted average of the correlation coefficients was then calculated using Formula (2) to obtain the weighted relational degree R.


(2) $$R_{0i}=\frac{1}{m}\sum_{k=1}^{m}W_{k}\cdot S\left(k\right)$$

## 3 Results and analysis

### 3.1 Differences in appearance quality of fruits from different mulberry varieties

The results regarding fruit weight, dimensions (length and width), shape index, moisture content, and color for various mulberry varieties are summarized in **Table 2**. Consumers primarily perceive fruit flavor through its visual appearance, color, texture, and aroma (Sean et al., 2020; Harry and Denise, 2018). The weight of individual fruits is related to the fruit’s yield, which in turn affects both its sales volume and price. Additionally, the fruit shape index allows for a direct assessment of the size and shape of the fruit, making it a key indicator of external quality.

**Table 2:** Differences in the appearance qualities of fruits in different varieties of mulberry trees.

Varieties	Single fruit heavy (g)	Transverse diameter (cm)	Longitudinal diameter (cm)	Fruit index	Water content(%)	Fruit shaped	Fruit color
Ri Ben Guo Sang	3.74 ± 0.06b	1.49 ± 0.04a	2.84 ± 0.02de	1.91 ± 0.04hij	86.00 ± 1.60bc	Cylindrical	Purple-Black
Jiang Mi Guo Sang	2.35 ± 0.06f	1.11 ± 0.06gh	2.33 ± 0.04gh	2.12 ± 0.15efg	77.41 ± 1.46fgh	Elongated Cylindrical	Pink-White
Tian Sang 202	1.99 ± 0.05ij	1.08 ± 0.04h	1.96 ± 0.05i	1.81 ± 0.10j	77.00 ± 1.17gh	Cylindrical	Jade-White
Hong Guo 1	3.68 ± 0.09b	1.45 ± 0.06ab	3.05 ± 0.14bc	2.11 ± 0.13efg	87.26 ± 1.13ab	Elongated Cylindrical	Purple-Red
Bai Shen 2	1.25 ± 0.15l	0.86 ± 0.01j	1.69 ± 0.06j	1.97 ± 0.08fghij	80.36 ± 1.38def	Cylindrical	Jade-White
Lv Shen Zi	4.03 ± 0.06a	1.30 ± 0.04cd	3.04 ± 0.13bc	2.34 ± 0.18d	83.04 ± 0.95cd	Elongated Cylindrical	Purple-Black
Lv Shen Zi 1	2.21 ± 0.09fh	1.12 ± 0.01gh	2.21 ± 0.04h	1.97 ± 0.03ghij	75.30 ± 0.22h	Cylindrical	Purple-Black
Lv Shen Zi 2	2.02 ± 0.08ij	1.02 ± 0.06i	2.31 ± 0.08gh	2.27 ± 0.05de	75.51 ± 0.96h	Elongated Cylindrical	Jade-White
Hei Zhen Zhu	3.12 ± 0.15d	1.40 ± 0.02b	2.77 ± 0.06e	1.98 ± 0.02fghij	82.14 ± 1.07de	Cylindrical	Purple-Black
Ji Gui Hua	2.30 ± 0.13f	1.08 ± 0.04h	2.24 ± 0.15h	2.07 ± 0.07fgh	75.45 ± 0.74h	Elongated Cylindrical	Jade-White
Gui Hua Mi	2.88 ± 0.23e	1.11 ± 0.02gh	2.93 ± 0.08cd	2.65 ± 0.09c	76.51 ± 1.04gh	Elongated Cylindrical	Pink-White
Xiao Bai E	3.50 ± 0.04c	1.16 ± 0.03fg	3.15 ± 0.05b	2.72 ± 0.13bc	79.19 ± 0.85efg	Elongated Cylindrical	Pink-White
Da Bai E	2.81 ± 0.03e	1.16 ± 0.00fg	2.72 ± 0.09e	2.35 ± 0.08d	76.17 ± 1.15gh	Elongated Cylindrical	Pink-White
Da Yi Bai	1.89 ± 0.08j	1.12 ± 0.02gh	2.40 ± 0.10fg	2.15 ± 0.10ef	75.49 ± 0.24h	Elongated Cylindrical	Jade-White
Su Bai Shen	2.76 ± 0.13e	1.33 ± 0.03c	2.54 ± 0.06f	1.91 ± 0.04hij	80.51 ± 0.63def	Cylindrical	Jade-White
Feng Guo Sang	1.67 ± 0.08k	1.11 ± 0.05gh	2.28 ± 0.10gh	2.05 ± 0.06fghi	82.41 ± 1.58de	Elongated Cylindrical	Purple-Black
He Lan Sang	3.65 ± 0.02bc	1.29 ± 0.01cd	3.02 ± 0.12bc	2.35 ± 0.08d	82.00 ± 0.89de	Elongated Cylindrical	Purple-Black
Ju Shen	3.71 ± 0.16b	1.21 ± 0.03ef	3.44 ± 0.05a	2.84 ± 0.11b	89.21 ± 0.37a	Elongated Cylindrical	Purple-Red
Da 10	2.08 ± 0.05hi	1.01 ± 0.04i	3.08 ± 0.07bc	3.07 ± 0.17a	81.84 ± 1.26de	Elongated Cylindrical	Purple-Black
Tang 10	2.82 ± 0.09e	1.26 ± 0.06de	2.94 ± 0.12cd	2.34 ± 0.02d	83.66 ± 1.07cd	Elongated Cylindrical	Purple-Black
Da Ma Ya	1.36 ± 0.12l	0.96 ± 0.03i	1.81 ± 0.01j	1.88 ± 0.06ij	68.37 ± 0.35i	Cylindrical	Jade-White

Values are expressed as mean ± standard deviation. Different lowercase letters indicate significant difference in Duncan test (*P*<0.05).

Among the 21 mulberry varieties, fruit shapes are primarily classified as cylindrical or elongated cylindrical, while colors are categorized into four groups: purple-black, pink-white, jade-white and purple-red. The average fruit weight ranges from 1.25 to 4.03 g, with ‘Lv Shen Zi’ exhibiting the highest weight at 4.03 g, significantly surpassing that of other varieties (*P*<0.05). ‘Ri Ben Guo Sang’ follows closely with a weight of 3.74 g. In contrast, ‘Bai Shen 2’ has a weight of only 1.25 g, comparable to ‘Da Ma Ya’, but significantly lower than that of most other varieties (*P*>0.05).

The fruit diameter across the various varieties ranges from 0.86 to 1.49 cm, while fruit length varies from 1.69 to 3.44 cm. ‘Ri Ben Guo Sang’ has the largest diameter at 1.49 cm, which is not significantly different from that of ‘Hong Guo 1’, while ‘Ju Shen’ boasts the longest fruit at 3.44 cm, significantly exceeding those of other varieties. Conversely, ‘Bai Shen 2’ exhibits the smallest dimensions, with a diameter of only 0.86 cm and a length of 1.69 cm; however, the latter measurement is not significantly different from that of ‘Da Ma Ya’.

The shape index for the 21 mulberry varieties ranges from 1.81 to 3.07, with ‘Da 10’ achieving the highest index of 3.07, significantly surpassing that of all other varieties. ‘Ju Shen’ follows closely with an index of 2.84, which is not significantly different from that of ‘Xiao Bai E’ but differs from other varieties. The shape index of ‘Tian Sang 202’ is comparable to those of ‘Hei Zhen Zhu’, ‘Bai Shen 2’, ‘Lv Shen Zi 1’, ‘Su Bai Shen’, ‘Ri Ben Guo Sang’ and ‘Da Ma Ya’; however, it is significantly lower than that of other varieties, at only 1.81.

Moisture content is a critical factor influencing the shelf life and processing characteristics of fruits (Yuan et al., 2022). Among the 21 mulberry varieties, moisture content ranges from 68.37% to 89.21%, with ‘Ju Shen’ exhibiting the highest level at 89.21%, which is not significantly different from that of ‘Hong Guo 1’ but is significantly higher than that of most other varieties. Conversely, ‘Da Ma Ya’ displays a significantly lower moisture content, recorded at only 68.37%.

The fruit shape index refers to the ratio of the longitudinal diameter to the transverse diameter of the fruit and is one of the quality indicators used to assess the fruit of many economic forest products. Relevant studies indicate that the quality of grape berries is significantly positively correlated with their length and diameter (Li et al., 2024a). In this study, the ‘Da Ma Ya’ variety had lower values for both individual fruit weight and fruit shape index, and its water content was also significantly lower than that of other varieties. Based on appearance quality, the fruit of the ‘Da Ma Ya’ variety was evaluated poorly. A comprehensive analysis of the above indicators showed that varieties such as ‘Ri Ben Guo Sang’, ‘Hong Guo 1’, ‘Lv Shen Zi’, ‘He Lan Sang’, ‘Ju Shen’, and ‘ Tang 10’ performed better in terms of individual fruit weight, fruit shape index, and water content. These varieties are primarily long-cylindrical in shape and range in color from dark purple to purplish-red, which visually enhances consumers’ purchasing desire and holds significant economic value.

### 3.2 Differences in nutritional quality of mulberry fruits from different varieties

The levels of free amino acids, titratable acids, soluble sugars, solid-acid ratios, soluble solids, and soluble proteins in fruits from various mulberry varieties are presented in **Table 3**.

**Table 3:** Differences in fruit nutritional quality of different varieties of mulberry trees.

Varieties	Free amino acid content (mg/g)	Titratable acid content (%)	Soluble sugar content (%)	Soluble solids to acidity ratio	Soluble solids content (%)	Soluble protein content(µg/g)	Tannin content (mg/g)
Ri Ben Guo Sang	228.93 ± 4.63c	1.58 ± 0.14de	7.83 ± 0.76h	5.02 ± 0.84ij	21.60 ± 2.07def	10.73 ± 0.25efg	2.24 ± 0.12ef
Jiang Mi Guo Sang	173.00 ± 7.09k	1.02 ± 0.11ij	20.50 ± 1.32c	20.20 ± 2.78cd	24.83 ± 1.16abcd	11.45 ± 0.70cde	0.42 ± 0.06ij
Tian Sang 202	198.96 ± 2.84ef	1.25 ± 0.10gh	24.33 ± 0.29b	19.46 ± 1.31de	24.08 ± 0.55bcde	10.88 ± 0.29defg	0.56 ± 0.07hij
Hong Guo 1	303.79 ± 7.51a	1.65 ± 0.08cd	12.00 ± 1.00g	7.29 ± 0.80ghi	13.01 ± 1.98g	12.19 ± 0.05abc	3.16 ± 0.02b
Bai Shen 2	214.73 ± 1.64d	1.36 ± 0.14fg	24.17 ± 0.76b	17.90 ± 1.46de	24.26 ± 20bcde	10.35 ± 0.23fg	0.37 ± 0.07j
Lv Shen Zi	219.58 ± 6.54d	1.65 ± 0.07cd	15.00 ± 1.00ef	9.09 ± 0.72g	22.56 ± 0.80cde	11.13 ± 0.20def	2.40 ± 0.19def
Lv Shen Zi 1	204.77 ± 4.96e	1.77 ± 0.14bcd	14.60 ± 0.36ef	8.31 ± 0.82gh	21.45 ± 1.36ef	11.44 ± 0.03cde	2.14 ± 0.08f
Lv Shen Zi 2	187.94 ± 2.19gh	1.64 ± 0.10cd	22.83 ± 1.61b	13.93 ± 0.52f	26.78 ± 2.10ab	11.44 ± 0.59cde	0.62 ± 0.09hij
Hei Zhen Zhu	192.67 ± 2.76fgh	1.81 ± 0.10bc	15.67 ± 1.26de	8.72 ± 1.15g	15.56 ± 1.18g	11.47 ± 0.61cde	2.46 ± 0.11de
Ji Gui Hua	184.27 ± 3.44hij	1.03 ± 0.06ij	23.50 ± 1.32b	22.97 ± 1.16b	23.15 ± 1.35cde	10.48 ± 0.38fg	0.58 ± 0.14hij
Gui Hua Mi	191.75 ± 0.91fgh	1.14 ± 0.08hij	20.33 ± 0.58c	17.91 ± 1.42de	24.33 ± 1.43bcde	10.80 ± 0.18efg	0.71 ± 0.03hi
Xiao Bai E	205.69 ± 6.34e	1.06 ± 0.10ij	23.17 ± 0.76b	21.97 ± 2.78bc	25.47 ± 1.22abc	10.87 ± 0.32defg	0.59 ± 0.08hij
Da Bai E	176.32 ± 1.76jk	0.78 ± 0.05k	26.50 ± 1.32a	33.99 ± 2.11a	27.64 ± 2.05a	11.50 ± 0.10cde	0.79 ± 0.10h
Da Yi Bai	187.86 ± 2.12gh	0.96 ± 0.08j	23.17 ± 1.26b	24.17 ± 0.66b	22.31 ± 2.55cde	10.87 ± 0.13defg	0.43 ± 0.20ij
Su Bai Shen	234.48 ± 2.95c	1.16 ± 0.02hi	17.17 ± 1.04d	14.74 ± 0.87f	22.33 ± 1.35cde	11.62 ± 0.82cd	0.33 ± 0.09j
Feng Guo Sang	176.41 ± 7.09jk	1.66 ± 0.08cd	13.17 ± 0.76fg	7.92 ± 0.46gh	21.43 ± 1.10ef	11.95 ± 0.49bc	1.56 ± 0.30g
He Lan Sang	177.67 ± 2.33ijk	1.82 ± 0.10bc	13.33 ± 1.53fg	7.37 ± 1.14ghi	23.35 ± 1.15cde	12.48 ± 0.80ab	3.48 ± 0.18a
Ju Shen	186.06 ± 3.57gh	2.53 ± 0.21a	15.67 ± 1.15de	6.23 ± 1.00hi	15.52 ± 2.22g	10.11 ± 0.29g	1.74 ± 0.25g
Da 10	194.67 ± 4.92fg	1.91 ± 0.06b	11.50 ± 1.80g	6.02 ± 0.84hi	21.19 ± 2.62ef	12.72 ± 0.33a	2.85 ± 0.31c
Tang 10	292.82 ± 8.46b	2.37 ± 0.12a	8.50 ± 0.50h	3.59 ± 0.11j	18.85 ± 1.28f	12.03 ± 0.18abc	2.53 ± 0.21d
Da Ma Ya	185.45 ± 2.32hi	1.46 ± 0.12ef	24.83 ± 0.29ab	17.09 ± 1.38e	21.37 ± 2.25ef	10.54 ± 0.22fg	0.69 ± 0.15hi

Values are expressed as mean ± standard deviation. Different lowercase letters indicate significant difference in Duncan test (*P*<0.05).

Free amino acids are readily absorbed by the human body, supporting metabolism, immune function, and growth, while also contributing to the diverse flavors of fruits (Shih et al., 2010). The content of free amino acids in the 21 mulberry varieties ranges from 173.00 to 303.79 mg/g. ‘Hong Guo 1’ exhibits the highest content at 303.79 mg/g, significantly surpassing other varieties. In contrast, ‘Jiang Mi Guo Sang’ contains 173.00 mg/g, which is not significantly different from that of ‘He Lan Sang’, ‘Feng Guo Sang’ and ‘Da Bai E’, yet is significantly lower than that of most other varieties.

The titratable acid content in fruits from various mulberry varieties ranges from 0.78% to 2.53%. ‘Ju Shen’ exhibits the highest titratable acid content at 2.53%, comparable to that of ‘Tang 10’, but significantly higher than that of other varieties. Conversely, ‘Da Bai E’ has the lowest titratable acid content at only 0.78%, significantly lower than that of other varieties. Chen et al. (Chen et al., 2022) reported titratable acid contents in mulberry fruits ranging from 0.397% to 7.479%, indicating a significant increase relative to the present study. Ange et al. noted a titratable acidity range of 0.93% to 2.65% for Spanish mulberries, which aligns closely with the findings of the current study (Angel et al., 2013).

Sugars are critical indicators of fruit sweetness and serve as the fundamental raw materials for synthesizing organic acids, pigments, and other nutritional components (Chen et al., 2015). Soluble sugars, primarily fructose, glucose, and sucrose, play a significant role in determining fruit sweetness, with fructose being the sweetest and glucose providing the most favorable taste (Dong et al., 2024). The soluble sugar content in fruits varies from 7.83% to 26.50%. ‘Da Bai E’ exhibits the highest soluble sugar content at 26.50%, with no significant difference from ‘Da Ma Ya’, whereas ‘Ri Ben Guo Sang’ displays a significantly lower content of only 7.83%.

The solid-acid ratio serves as a critical indicator for assessing fruit flavor and ripeness, with a higher ratio signifying sweeter fruit (Huang et al., 2021). Among the 21 varieties, this ratio varies from 3.59 to 33.99, with ‘Da Bai E’ exhibiting the highest value at 33.99, significantly surpassing all other varieties. ‘Da Yi Bai’ and ‘Ji Gui Hua’ follow; however, both are significantly lower than ‘Da Bai E’. The solid-acid ratio for ‘Tang 10’ is not significantly different from that of ‘Ri Ben Guo Sang’; however, it is significantly lower than that of other varieties, at only 3.59.

The soluble solids content in fruits varies from 13.01% to 27.64%. ‘Da Bai E’ exhibits the highest soluble solids content at 27.64%, followed closely by ‘Lv Shen Zi 2’, ‘Xiao Bai E’ and ‘Jiang Mi Guo Sang’, with no significant differences among these varieties. ‘Hong Guo 1’ has a soluble solids content of only 13.01%, which showed no significant difference compared to ‘Hei Zhen Zhu’ and ‘Ju Shen’, but it was significantly lower than other varieties.

Soluble protein content is another crucial indicator of fruit and vegetable quality and nutritional value. The soluble protein content in the different mulberry varieties ranges from 10.11 to 12.72 µg/g. ‘Da 10’ exhibits the highest soluble protein content at 12.72 µg/g, which is not significantly different from that of ‘He Lan Sang’, ‘Hong Guo 1’ and ‘Tang 10’, yet is significantly higher than other varieties. ‘Ju Shen’ contains the lowest soluble protein content at only 10.11 µg/g. Studies have shown that soluble proteins contain essential amino acids and peptides, which have health benefits including antibacterial, antioxidant and anti-inflammatory properties (Kuldip et al., 2024), and the relatively high protein content of mulberries also enhances their suitability as functional foods.

A comprehensive understanding of these nutritional factors, along with varietal differences, can assist both consumers and producers in selecting the most suitable mulberry varieties for specific applications, including fresh consumption, functional foods, and processed products. This study focused on analyzing nutritional qualities, including free amino acids, titratable acids, soluble sugars, solid acid ratio, and soluble proteins. Based on the analysis of these nutritional qualities, the mulberries of the ‘Da Bai E’ and ‘Da Yi Bai’ varieties exhibit relatively superior nutritional quality. Among these, ‘Da Bai E’ has the highest solid acid ratio, making it an ideal choice for fresh consumption. However, both varieties have comparatively low free amino acid content. The ‘He Lan Sang’ and ‘Tang 10’ varieties are comparatively inferior, with higher titratable acid content and lower solid acid ratios. In contrast, the ‘Tang 10’ variety exhibits slightly higher free amino acid content.

### 3.3 Differences in functional components of mulberry fruits from different varieties

The concentrations of carotenoids, flavonoids, polyphenols, anthocyanins, resveratrol, chlorogenic acid, ascorbic acid, and tannins in fruits from various mulberry varieties are summarized in **Table 4**.

**Table 4:** Differences in the functional composition of fruits in different varieties of mulberry trees.

Varieties	Carotenoid content (mg/g)	Flavonoid content (mg/g)	Polyphenol content (mg/g)	Anthocyanin (mg/g)	Resveratrol content (mg/g)	Chlorogenic acid content (mg/g)	Ascorbic acid content (mg/100g)
Ri Ben Guo Sang	0.39 ± 0.01de	88.78 ± 4.74a	148.37 ± 5.67a	39.35 ± 2.81g	2.63 ± 0.02ab	41.36 ± 0.80a	382.76 ± 57.95ef
Jiang Mi Guo Sang	0.22 ± 0.01gh	10.43 ± 0.90f	17.02 ± 1.03i	2.75 ± 0.08i	0.39 ± 0.03gh	7.30 ± 0.25g	202.31 ± 34.71ghi
Tian Sang 202	0.24 ± 0.03gh	10.97 ± 0.90f	13.87 ± 1.86ij	0.28 ± 0.10i	0.24 ± 0.00h	7.93 ± 0.84g	116.12 ± 24.35jk
Hong Guo 1	0.93 ± 0.08a	41.57 ± 0.79e	118.40 ± 8.38d	32.34 ± 1.74h	2.39 ± 0.13c	31.36 ± 1.18c	386.77 ± 57.02ef
Bai Shen 2	0.21 ± 0.04h	10.09 ± 0.43f	23.49 ± 1.79h	0.31 ± 0.09i	0.08 ± 0.01i	7.30 ± 0.32g	129.70 ± 23.33jk
Lv Shen Zi	0.52 ± 0.01c	69.30 ± 4.08c	129.68 ± 3.64c	42.08 ± 0.17ef	2.39 ± 0.07c	35.81 ± 2.18b	661.59 ± 56.49c
Lv Shen Zi 1	0.61 ± 0.11b	54.79 ± 1.90d	122.28 ± 7.41d	43.86 ± 1.27e	2.15 ± 0.04d	29.46 ± 2.36d	493.13 ± 52.09d
Lv Shen Zi 2	0.32 ± 0.08efgh	11.91 ± 1.82f	14.45 ± 1.66ij	1.05 ± 0.13i	0.29 ± 0.02h	7.70 ± 0.24g	111.36 ± 2.57jk
Hei Zhen Zhu	0.67 ± 0.10b	64.84 ± 1.97c	124.23 ± 5.25cd	53.66 ± 2.01c	1.80 ± 0.16e	31.57 ± 1.30c	424.90 ± 30.97e
Ji Gui Hua	0.24 ± 0.03gh	13.41 ± 1.07f	49.35 ± 3.25f	0.20 ± 0.03i	0.71 ± 0.04f	7.44 ± 0.39g	137.15 ± 4.56ijk
Gui Hua Mi	0.23 ± 0.06gh	14.36 ± 1.00f	32.29 ± 1.72g	0.26 ± 0.15i	0.80 ± 0.06f	6.89 ± 0.09g	172.73 ± 9.85hij
Xiao Bai E	0.06 ± 0.03i	9.65 ± 0.52f	18.16 ± 0.47hi	0.44 ± 0.04i	0.37 ± 0.03gh	15.08 ± 0.27f	165.76 ± 16.90hij
Da Bai E	0.03 ± 0.01i	11.79 ± 0.23f	13.44 ± 1.29ij	0.33 ± 0.25i	0.31 ± 0.09gh	17.23 ± 0.04e	166.37 ± 11.25hij
Da Yi Bai	0.24 ± 0.06gh	8.70 ± 1.64f	12.27 ± 1.33ij	0.34 ± 0.19i	0.36 ± 0.03gh	7.22 ± 0.23g	107.57 ± 14.91jk
Su Bai Shen	0.31 ± 0.03efgh	9.19 ± 0.68f	8.42 ± 0.57j	0.26 ± 0.04i	0.24 ± 0.06h	6.85 ± 0.48g	72.36 ± 0.73k
Feng Guo Sang	0.33 ± 0.11efg	80.00 ± 6.68b	150.24 ± 1.92a	56.33 ± 2.77b	2.50 ± 0.08bc	32.64 ± 1.01c	330.54 ± 15.04ef
He Lan Sang	0.36 ± 0.04def	67.72 ± 7.85c	145.83 ± 2.48a	55.66 ± 1.68bc	2.16 ± 0.16d	32.56 ± 1.23c	887.93 ± 72.81a
Ju Shen	0.49 ± 0.03c	58.23 ± 10.20d	136.90 ± 3.83b	41.36 ± 1.84fg	2.24 ± 0.05d	29.48 ± 0.76d	528.44 ± 42.36d
Da 10	0.44 ± 0.04cd	90.45 ± 5.78a	145.66 ± 3.13a	60.62 ± 2.31a	2.57 ± 0.10ab	34.71 ± 1.84b	760.63 ± 61.01b
Tang 10	0.31 ± 0.04efgh	38.45 ± 2.10e	97.87 ± 3.27e	48.26 ± 1.86d	2.67 ± 0.15a	18.46 ± 0.74e	252.32 ± 15.59f
Da Ma Ya	0.28 ± 0.04fgh	8.37 ± 0.90f	8.71 ± 0.55j	0.52 ± 0.35i	0.45 ± 0.06g	8.70 ± 0.23g	225.54 ± 59.88g

Values are expressed as mean ± standard deviation. Different lowercase letters indicate significant difference in Duncan test (P<0.05).

The main carotenoids found in fruits are β-carotene. These carotenoids are crucial for human health, particularly in preventing age-related macular degeneration and supporting overall eye health (Thomas et al., 2024). The carotenoid content in fruits from 21 mulberry varieties varies from 0.03 to 0.93 mg/g. ‘Hong Guo 1’ exhibits the highest carotenoid content at 0.93 mg/g, significantly exceeding that of other varieties. ‘Hei Zhen Zhu’ and ‘Lv Shen Zi 1’ follow, with contents of 0.67 and 0.61 mg/g, respectively. In contrast, ‘Da Bai E’ has a carotenoid content of merely 0.03 mg/g, which is significantly lower than that of other varieties, but there was no significant difference from ‘Xiao Bai E’.

Flavonoids possess significant antioxidant properties and serve as precursors for flavor compounds, rendering them one of the primary phenolic compounds in dietary extracts (Chang et al., 2024). The flavonoid content in fruits ranges from 8.37 to 90.45 mg/g, with an average of 36.81 mg/g. ‘Da 10’ exhibits the highest flavonoid content at 90.45 mg/g, which is not significantly different from that of ‘Ri Ben Guo Sang’, but significantly higher than that of other varieties. Conversely, ‘Da Ma Ya’ contains the lowest flavonoid content at 8.37 mg/g. Ireneusz et al. (Ireneusz et al., 2015) analyzed the flavonoid content in various cultivated blueberry varieties, reporting a range of 5.63 to 22.10 mg/g, which is lower than that observed in this study.

Research indicates that phenolic compounds in grapes may help prevent disorders associated with obesity-induced dyslipidemia and regulate estrogen levels and platelet aggregation (Li et al., 2019; Elenara et al., 2019). Priti et al. (Priti et al., 2023) found that the polyphenol content in blueberries ranges from 48 to 304 mg/100 g of fresh fruit weight. In this study, the polyphenol content in mulberry fruits ranges from 8.42 to 150.24 mg/g, with an average of 72.90 mg/g, which is relatively high in comparison to that of blueberries. ‘Feng Guo Sang’ exhibits the highest polyphenol content at 150.24 mg/g. The polyphenol content of ‘Su Bai Shen’ had no significant difference from that of ‘Da Ma Ya’, but had no significant difference from that of ‘Lv Shen Zi 2’, ‘Tian Sang 202’, ‘Da Bai E’ and ‘Da Yi Bai’, which was significantly lower than that of other varieties. at only 8.42 mg/g. Yim et al. reported that the total phenol content in 10 pear varieties ranges from 135.2 to 161.2 mg/100 g (Yim and Nam, 2016). Related studies indicate that most varieties from organic berry plantations are characterized by high levels of polyphenolic compounds, with an average content of 288 mg/100 g, which is higher compared to this study; this may suggest that organic soil enhances the content of phenolic compounds (Ireneusz et al., 2015).

Anthocyanins, a major class of secondary metabolites, play essential nutritional and pharmacological roles, attracting increasing attention (Chen et al., 2022). The anthocyanin content in fruits from different mulberry varieties ranges from 0.20 to 60.62 mg/g, with an average of 22.87 mg/g, indicating significant variation. ‘Da 10’ exhibits the highest anthocyanin content at 60.62 mg/g, significantly exceeding that of other varieties. The anthocyanin content in ‘Ji Gui Hua’ was the lowest, showing no significant difference compared to ‘Jiang Mi Guo Sang’, ‘Lv Shen Zi 2’, ‘Da Ma Ya’, ‘Xiao Bai E’, ‘Da Yi Bai’ and ‘Da Bai E’, at only 0.20 mg/g. In related studies, the primary anthocyanin content was considered as the sum of three types of anthocyanins (C3G, C3R, P3G), detected by high-performance liquid chromatography, ranging from 0.882 mg/g to 5.737 mg/g (Chen et al., 2022).

Resveratrol is a significant polyphenol in mulberries, recognized for its beneficial effects against viruses, cardiovascular diseases, inflammation, and cancer (Yusuf et al., 2015; Shunsuke et al., 2009; Jang et al., 1997). The resveratrol content in fruits from the 21 varieties ranges from 0.08 to 2.67 mg/g, with an average of 1.32 mg/g. ‘Tang 10’ exhibits the highest resveratrol content at 2.67 mg/g, which is not significantly different from that of ‘Ri Ben Guo Sang’ and ‘Da 10’, but is significantly higher than that of other varieties. ‘Bai Shen 2’ contains the lowest resveratrol content at only 0.08 mg/g.

Chlorogenic acid, a prominent phenolic compound, possesses antioxidant, antibacterial, anticancer, and UV-protective properties (He et al., 2017). The chlorogenic acid content in fruits varies from 6.85 to 41.36 mg/g, with ‘Ri Ben Guo Sang’ exhibiting the highest content at 41.36 mg/g, significantly higher than that of other varieties. ‘Lv Shen Zi’ and ‘Da 10’ follow in terms of chlorogenic acid content. The chlorogenic acid content in ‘Su Bai Shen’ showed no significant difference compared to ‘Tian Sang 202’, ‘Lv Shen Zi 2’, ‘Ji Gui Hua’, ‘Bai Shen 2’, ‘Jiang Mi Guo Sang’, ‘Da Yi Bai’ and ‘Gui Hua Mi’, but it was significantly lower than other varieties, at only 6.85 mg/g.

Ascorbic acid is crucial for maintaining a healthy immune system, promoting wound healing, and preventing oxidative stress. Its antioxidant properties help protect the body from damage caused by free radicals and make it an important nutrient for skin health (Bisma et al., 2021). The ascorbic acid content in fruits from various mulberry varieties ranges from 72.36 to 887.93 mg/100 g. ‘He Lan Sang’ possesses the highest ascorbic acid content at 887.93 mg/100 g, significantly exceeding that of other varieties. The ascorbic acid content in ‘Su Bai Shen’ showed no significant difference compared to ‘Ji Gui Hua ‘, ‘Bai Shen 2’, ‘Tian Sang 202’, ‘Lv Shen Zi 2’ and ‘Da Yi Bai’, but it was significantly lower than other varieties, at only 72.36 mg/100 g.

Tannins, which are secondary metabolites commonly found in many plants, contribute to the astringency of fruits (Li et al., 2024). The tannin content across different mulberry varieties ranges from 0.33 to 3.48 mg/g, with ‘He Lan Sang’ exhibiting the highest content at 3.48 mg/g, significantly higher than that of other varieties. ‘Su Bai Shen’ has a tannin content comparable to that of ‘Bai Shen 2’, ‘Lv Shen Zi 2’, ‘Xiao Bai E’, ‘Ji Gui Hua’, ‘Tian Sang 202’, ‘Da Yi Bai’ and ‘Jiang Mi Guo Sang’, yet is significantly lower than other varieties at only 0.33 mg/g.

Significant variations exist in the content of bioactive compounds—such as carotenoids, flavonoids, polyphenols, anthocyanins, resveratrol, chlorogenic acid, ascorbic acid, and tannins—across different mulberry varieties. A comprehensive analysis of the functional components of these varieties reveals that varieties such as ‘Da 10’, ‘Ri Ben Guo Sang’, and ‘Feng Guo Sang’ exhibit superior overall performance, with each indicator standing out. In contrast, the ‘Su Bai Shen’ and ‘Da Yi Bai’ varieties perform poorly across all indicators, resulting in a slightly lower overall evaluation. Understanding these varietal differences is essential for selecting the most advantageous mulberry varieties for functional foods, nutritional supplements, and health products.

### 3.4 Differences in antioxidant quality of mulberry fruits from different varieties

#### 3.4.1 Differences in antioxidant capacity among different mulberry varieties

The antioxidant capacities of mulberry fruits from various varieties, including DPPH radical scavenging ability, ABTS radical scavenging ability, iron reduction capacity, and MDA content, are summarized in **Table 5**.

**Table 5:** Differences in the oxidation resistance of fruits in different varieties of mulberry trees.

Varieties	DPPH free radical scavenging ability µmol/L (Trolox)	DPPH free radical scavenging ability µmol/L (Trolox)	Iron reduction capability (µmol/L) Trolox	MDA content(µmol/g)
Ri Ben Guo Sang	22.67 ± 1.33k	20.51 ± 0.14cd	27.03 ± 0.63a	30.79 ± 1.39d
Jiang Mi Guo Sang	38.37 ± 1.13ab	20.87 ± 0.25abc	1.59 ± 0.76g	8.13 ± 4.31e
Tian Sang 202	30.89 ± 2.47ef	20.83 ± 0.47abc	1.14 ± 0.32g	4.50 ± 1.06e
Hong Guo 1	32.31 ± 1.69de	20.54 ± 0.13cd	13.33 ± 1.87e	56.12 ± 3.08c
Bai Shen 2	31.50 ± 0.19def	20.78 ± 0.18abc	0.49 ± 0.23g	8.23 ± 3.45e
Lv Shen Zi	25.05 ± 0.20ij	20.83 ± 0.18abc	20.19 ± 1.59bcd	113.95 ± 6.52a
Lv Shen Zi 1	27.52 ± 1.39gh	21.03 ± 0.06ab	19.39 ± 0.58cd	27.16 ± 3.52d
Lv Shen Zi 2	22.86 ± 0.92k	19.90 ± 0.22e	1.66 ± 0.45g	6.44 ± 0.67e
Hei Zhen Zhu	29.48 ± 1.20fg	21.01 ± 0.24ab	18.05 ± 1.32d	74.28 ± 14.47b
Ji Gui Hua	39.69 ± 0.15ab	21.03 ± 0.22ab	9.72 ± 1.54f	5.87 ± 1.93e
Gui Hua Mi	40.26 ± 0.39a	20.66 ± 0.25bcd	10.44 ± 4.10ef	6.35 ± 1.14e
Xiao Bai E	37.83 ± 0.80b	20.29 ± 0.31de	2.05 ± 0.33g	7.43 ± 1.14e
Da Bai E	34.49 ± 0.98c	21.01 ± 0.08ab	1.47 ± 0.75g	6.18 ± 1.68e
Da Yi Bai	31.00 ± 0.33ef	21.15 ± 0.16a	1.68 ± 0.87g	5.35 ± 1.04e
Su Bai Shen	11.90 ± 0.74l	21.07 ± 0.18ab	1.20 ± 0.44g	5.89 ± 1.36e
Feng Guo Sang	23.95 ± 0.84jk	20.84 ± 0.28abc	22.77 ± 2.85b	112.26 ± 17.02a
He Lan Sang	26.93 ± 0.43hi	20.82 ± 0.13abc	17.87 ± 1.54d	71.06 ± 23.37b
Ju Shen	27.81 ± 1.97gh	21.04 ± 0.10ab	18.17 ± 2.31d	55.34 ± 1.66c
Da 10	24.08 ± 0.83jk	20.05 ± 0.13e	22.00 ± 2.40bc	80.62 ± 18.10b
Tang 10	33.48 ± 2.06cd	20.06 ± 0.31e	12.76 ± 3.77ef	81.55 ± 5.40b
Da Ma Ya	30.84 ± 0.31ef	20.90 ± 0.06abc	2.47 ± 0.55g	6.24 ± 1.04e

Values are expressed as mean ± standard deviation. Different lowercase letters indicate significant difference in Duncan test (*P*<0.05).

High DPPH scavenging capacity indicates the ability to effectively neutralize free radicals, thereby reducing the risk of oxidative stress and chronic inflammatory-related diseases, such as cardiovascular diseases and cancer (Liu and Zhang, 2024). The DPPH radical scavenging ability of fruits from 21 mulberry varieties varies from 11.90 to 40.26 µmol/L (Trolox), with an average of 29.67 µmol/L (Trolox). ‘Gui Hua Mi’ exhibits the highest DPPH radical scavenging ability at 40.26 µmol/L (Trolox), significantly surpassing other varieties. ‘Ji Gui Hua’ and ‘Jiang Mi Guo Sang’ follow with elevated values, significantly exceeding those of other varieties. ‘Su Bai Shen’ exhibits the lowest DPPH radical scavenging ability at 11.90 µmol/L (Trolox), significantly lower than that of other varieties. Significant differences in DPPH scavenging ability were observed among 10 pear varieties, with bleaching ability ranging from 20.5% to 58.8% (Yim and Nam, 2016). In the study by Catarina et al (Catarina et al., 2018), the peach variety Royal Lu exhibited the strongest DPPH radical scavenging ability, with an IC50 of 62.0 µg/mL, closely associated with phenolic compounds. In this experiment, the ability to scavenge DPPH free radicals ranged from 11.90 to 40.26 µmol/L, indicating a relatively weak capacity.

The ABTS radical scavenging ability of fruits from various mulberry varieties varies from 19.90 to 21.15 µmol/L (Trolox), with an average of 20.72 µmol/L (Trolox). ‘Da Yi Bai’ demonstrates the highest ABTS radical scavenging ability at 21.15 µmol/L (Trolox). ‘Lv Shen Zi 2’ exhibits the lowest ABTS radical scavenging ability at 19.90 µmol/L (Trolox), comparable to ‘Xiao Bai E’, ‘Tang 10’ and ‘Da 10’, but significantly lower than that of other varieties.

The iron-reducing capacity of fruits serves as a key indicator of their overall antioxidant potential. It helps prevent the formation of hydroxyl radicals, which are highly reactive and contribute to oxidative damage. By enhancing the body’s antioxidant defense system, it may reduce the risk of oxidative stress-related diseases, including atherosclerosis and diabetes (Yohanes et al., 2020). The iron-reducing capacity of fruits from various mulberry varieties ranges from 0.49 to 27.03 µmol/L (Trolox), with an average of 10.74 µmol/L (Trolox). ‘Ri Ben Guo Sang’ exhibits the highest iron-reducing capacity at 27.03 µmol/L (Trolox), significantly surpassing all other varieties. ‘Feng Guo Sang’ follows with a capacity of 22.77 µmol/L (Trolox), which is not significantly different from that of ‘Da 10’ and ‘Lv Shen Zi’, but is significantly higher than that of the remaining varieties. ‘Bai Shen 2’ exhibits the lowest iron-reducing capacity at 0.49 µmol/L (Trolox).

The formation of MDA is commonly used as an indicator of oxidative stress and tissue damage. The MDA content in fruits from various mulberry varieties varies from 4.50 to 113.95 µmol/g, with an average of 36.84 µmol/g. ‘Lv Shen Zi’ exhibits the highest MDA content at 113.95 µmol/g, showing no significant difference compared to ‘Feng Guo Sang’, but it was significantly higher than other varieties. ‘Tang 10’ exhibits MDA content similar to that of ‘Da 10’, ‘Hei Zhen Zhu’ and ‘He Lan Sang’, all of which exceed the average. ‘Tian Sang 202’ demonstrates the lowest MDA content at 4.50 µmol/g.

Based on the results above, including measurements of DPPH, ABTS, iron-reducing capacity, and malondialdehyde content, ‘Gui Hua Mi’ exhibits exceptional DPPH radical scavenging ability. Overall, varieties such as ‘Feng Guo Sang’, ‘Lv Shen Zi’ and ‘Hei Zhen Zhu’ display robust antioxidant capabilities, while ‘Su Bai Shen’, and ‘Lv Shen Zi 2’ exhibit diminished antioxidant activity.

#### 3.4.2 Differences in enzyme activity among mulberry fruit varieties

Fruits with high antioxidant activity demonstrate superior storage and transportation characteristics, enhanced resistance to adverse conditions, and offer significant health benefits. Consequently, there is growing interest in research regarding the antioxidant activity of fruits (Park et al., 2020; Shiferaw et al., 2020). The activities of SOD, POD, CAT, polyphenol oxidase (PPO), and lipoxygenase (LOX) in fruits from various mulberry varieties are presented in **Table 6**.

**Table 6:** Differences in fruit enzyme activity in different varieties of mulberry trees (U·min·g^-1^FW).

Varieties	SOD activity	POD activity	CAT activity	PPO activity	LOX activity
Ri Ben Guo Sang	19.97 ± 0.78defg	16.92 ± 1.96bcd	36.33 ± 3.64cd	129.44 ± 11.66ab	168.00 ± 10.39de
Jiang Mi Guo Sang	27.17 ± 1.16a	14.35 ± 1.12de	39.50 ± 4.59bc	46.80 ± 5.77jk	124.00 ± 34.12e
Tian Sang 202	21.57 ± 0.81cde	19.08 ± 0.67ab	30.33 ± 6.48def	77.20 ± 19.8fghi	138.00 ± 43.27e
Hong Guo 1	23.23 ± 2.16bc	20.59 ± 1.66a	37.92 ± 2.92cd	81.76 ± 9.17efg	152.00 ± 81.46e
Bai Shen 2	18.74 ± 2.06efg	5.88 ± 1.80k	27.50 ± 1.52efg	75.28 ± 9.62fghij	258.00 ± 111.12bcd
Lv Shen Zi	20.58 ± 2.70cdefg	12.5 ± 0.52ef	34.67 ± 4.06cde	83.28 ± 8.66ef	176.00 ± 22.72de
Lv Shen Zi 1	19.57 ± 1.69efg	12.65 ± 2.52ef	69.58 ± 6.85a	119.20 ± 4.86bc	126.00 ± 15.87e
Lv Shen Zi 2	18.28 ± 1.64fg	9.14 ± 0.75hij	36.17 ± 3.26cd	140.24 ± 13.85a	284.00 ± 52.42bc
Hei Zhen Zhu	26.81 ± 1.16a	8.45 ± 1.22ij	12.25 ± 1.75ij	37.36 ± 8.93k	330.00 ± 49.11b
Ji Gui Hua	20.54 ± 2.04cdefg	8.88 ± 1.47hij	36.58 ± 2.67cd	55.84 ± 10.71ijk	204.00 ± 84cde
Gui Hua Mi	19.55 ± 2.35efg	12.53 ± 1.83ef	30.25 ± 3.44def	62.88 ± 7.28ghij	200.00 ± 54.11cde
Xiao Bai E	17.72 ± 0.28g	12.20 ± 1.00efg	30.75 ± 2.18def	60.00 ± 9.5hij	128.00 ± 12.49e
Da Bai E	21.6 ± 2.33cde	11.49 ± 1.13fgh	34.00 ± 7.60cde	65.68 ± 6.39fghij	174.00 ± 53.33de
Da Yi Bai	20.94 ± 1.36cdef	9.76 ± 0.94ghi	12.25 ± 3.61ij	99.20 ± 16.46de	140.00 ± 6.93e
Su Bai Shen	23.07 ± 1.35bcd	15.50 ± 2.63d	22.42 ± 3.79gh	53.84 ± 15.72jk	170.00 ± 56.71de
Feng Guo Sang	20.82 ± 1.86cdefg	10.21 ± 2.03fghi	19.33 ± 2.02hi	7.04 ± 3.00l	516.00 ± 102.53a
He Lan Sang	26.40 ± 1.26a	10.89 ± 0.53fghi	35.42 ± 4.54cde	122.56 ± 16.10ab	142.00 ± 24.25e
Ju Shen	25.08 ± 1.18ab	6.81 ± 0.76jk	10.75 ± 2.46j	60.80 ± 3.06hij	184.00 ± 30.79de
Da 10	24.93 ± 1.74ab	18.67 ± 1.02abc	45.83 ± 6.93b	57.44 ± 12.53hij	170.00 ± 55.75de
Tang 10	21.73 ± 1.41cde	16.26 ± 1.24cd	25.75 ± 3.25fgh	11.92 ± 3.50l	152.00 ± 15.1e
Da Ma Ya	18.31 ± 1.19fg	14.36 ± 1.36de	31.08 ± 3.50def	103.68 ± 8.65cd	122.00 ± 21.07e

Values are expressed as mean ± standard deviation. Different lowercase letters indicate significant difference in Duncan test P<0.05.

In the process of free radical scavenging, the initial antioxidant enzyme to act is superoxide dismutase (SOD). SOD rapidly dismutates superoxide anions (O2-) into hydrogen peroxide (H2O2) and molecular oxygen (Hasanuzzaman et al., 2019). Although H₂O₂ is harmful, it is further decomposed into harmless water by catalase (CAT) and peroxidase (POD). Collectively, SOD, POD, and CAT constitute an antioxidant system that maintains a dynamic balance of reactive oxygen species in plants (Wang et al., 2021; Yilmaz et al., 2017). The SOD activity in fruits from 21 mulberry varieties ranges from 17.72 to 27.17 U·min·g⁻¹ FW, with a mean value of 21.74 U·min·g⁻¹ FW. ‘Jiang Mi Guo Sang’ exhibits the highest SOD activity at 27.17 U·min·g⁻¹ FW, whereas ‘Xiao Bai E’ demonstrates the lowest SOD activity at 17.72 U·min·g⁻¹ FW.

The increase in POD activity also contributes to the anti-inflammatory and antioxidant effects of the fruit, which is beneficial for overall health, including reducing the risk of chronic diseases such as diabetes, cancer, and heart disease (Wang et al., 2021). POD activity in fruits from various mulberry varieties ranges from 5.88 to 20.59 U·min·g⁻¹ FW, with a mean value of 12.72 U·min·g⁻¹ FW. ‘Hong Guo 1’ exhibits the highest POD activity at 20.59 U·min·g⁻¹ FW. ‘Bai Shen. 2’ demonstrates the lowest POD activity at 5.88 U·min·g⁻¹ FW. Nine varieties, including ‘Tian Sang 202’, ‘Da 10’, ‘Ri Ben Guo Sang’ and ‘Tang 10’, exhibit POD activities exceeding the mean value.

Catalase (CAT) plays a crucial role in the detoxification of hydrogen peroxide, thereby mitigating oxidative damage and preventing cellular aging and tissue degeneration (Wang et al., 2021). CAT activity in fruits from various mulberry varieties ranges from 10.75 to 69.58 U·min·g⁻¹ FW, with a mean value of 31.37 U·min·g⁻¹ FW. ‘Lv Shen Zi 1’ exhibits the highest CAT activity at 69.58 U·min·g⁻¹ FW, significantly surpassing that of all other varieties. Higher CAT activity suggests more effective protection of cells from oxidative damage and a reduced risk of diseases linked to chronic inflammation. ‘Da 10’ exhibits the next highest CAT activity at 45.83 U·min·g⁻¹ FW. ‘Ju Shen’ exhibits CAT activity comparable to that of ‘Hei Zhen Zhu’ and ‘Da Yi Bai’, but significantly lower than that of other varieties, measuring only 10.75 U·min·g⁻¹ FW.

PPO activity in fruits can affect their taste, color, texture, and oxidative stability. However, excessive PPO activity can lead to nutrient loss and a decrease in fruit quality (Wang et al., 2017). PPO activity in fruits from various mulberry varieties ranges from 7.04 to 140.24 U·min·g⁻¹ FW, with a mean value of 73.88 U·min·g⁻¹ FW. ‘Lv Shen Zi 2’ demonstrates the highest PPO activity at 140.24 U·min·g⁻¹ FW. ‘Feng Guo Sang’ exhibits PPO activity comparable to that of ‘Tang 10’, but is significantly lower than that of other varieties, measuring only 7.04 U·min·g⁻¹ FW. The higher PPO activity in mulberries may be due to their lower anthocyanin content, which makes them more susceptible to enzymatic browning.

LOX activity in fruits from various mulberry varieties ranges from 122.00 to 516.00 U·min·g⁻¹ FW, with a mean value of 193.24 U·min·g⁻¹ FW. ‘Feng Guo Sang’ demonstrates the highest LOX activity at 516.00 U·min·g⁻¹ FW, significantly surpassing other varieties. ‘Da Ma Ya’ exhibits the lowest LOX activity at 122.00 U·min·g⁻¹ FW. The lower LOX activity in mulberries may be related to their higher content of anthocyanins and other antioxidants, which reduces the need for LOX-induced oxidative processes.

Overall, ‘Da 10’ demonstrates robust enzyme activities in superoxide dismutase (SOD), peroxidase (POD), and catalase (CAT), whereas the ‘Lv Shen Zi 2’ variety exhibits high polyphenol oxidase (PPO) enzyme activity. ‘Ju Shen’ generally exhibits considerable SOD activity; however, its activities for the remaining four enzymes are comparatively weak, falling below the average. Varieties such as ‘Jiang Mi Guo Sang’, ‘Hong Guo 1’, and ‘He Lan Sang’ demonstrate high antioxidant enzyme activity and low lipoxygenase activity, indicating strong resistance to oxidative stress and reduced lipid peroxidation. These characteristics make them particularly beneficial in mitigating the risk of inflammation, aging, and chronic diseases.

### 3.5 Comprehensive evaluation of fruit quality among different mulberry varieties

#### 3.5.1 Correlation analysis

**Figure 1:**
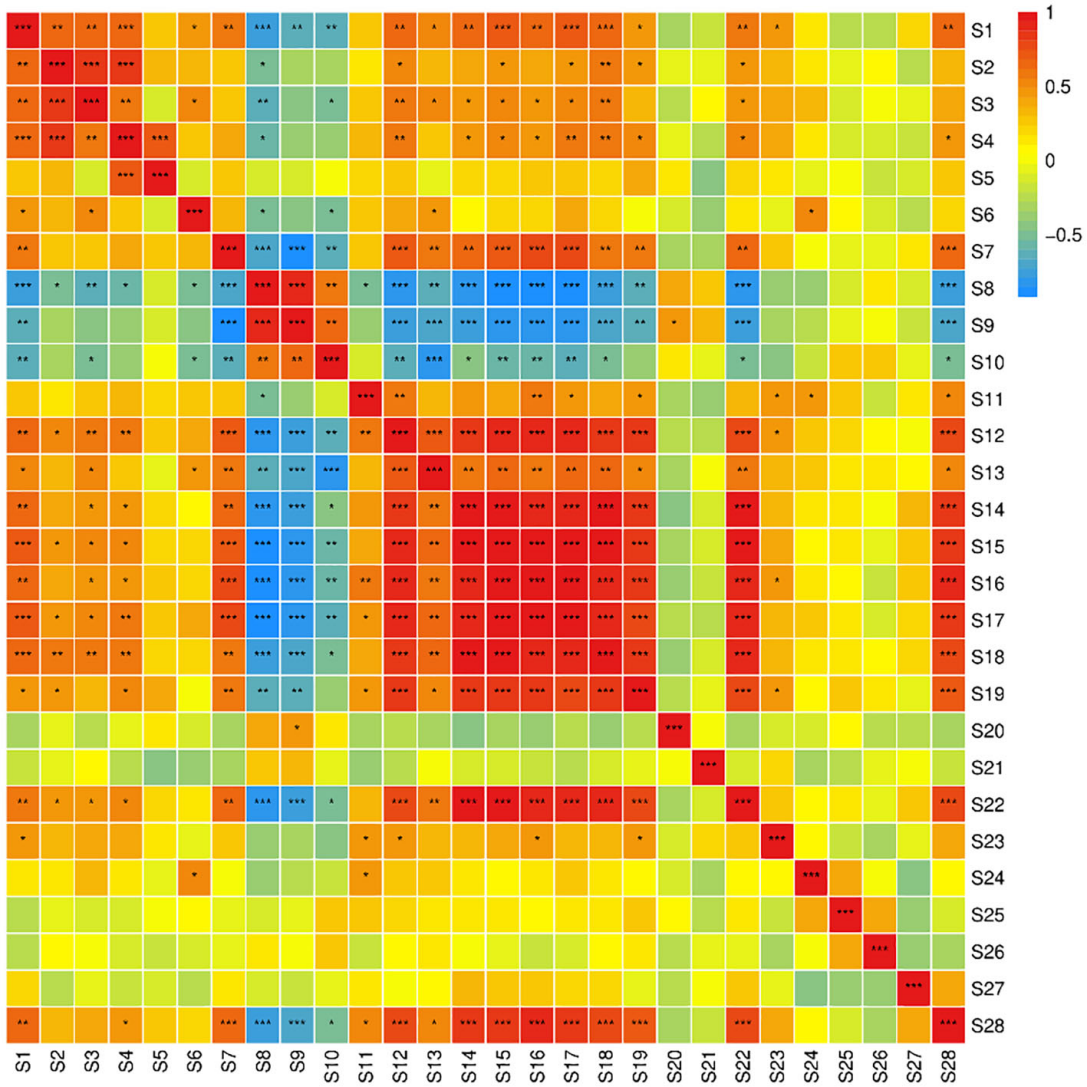
Correlation analysis of mulberry fruit quality traits. S1-water content, S2-single fruit weight, S3-fruit transverse diameter, S4-fruit longitudinal diameter, S5-fruit shape index, S6-free amino acid content, S7-titratable acid content, S8-soluble sugar content, S9-solid acid ratio, S10-soluble solid content, S11-soluble protein content, S12-tannin content, S13-carotenoid content, S14-flavonoid content, S15-polyphenol content, S16-anthocyanin content, S17-Resveratrol content, S18-chlorogenic acid content, S19-ascorbic acid content, S20-DPPH free radical scavenging ability, S21-ABTS free radical scavenging ability, S22-iron reducing ability, S23-SOD activity, S24-POD activity, S25-CAT activity, S26-PPO activity, S27-LOX activity, S28-MDA content. the same below.

The results of the Pearson correlation analysis for 28 indicators of fruit quality traits across 21 mulberry varieties (**Figure 1**) reveal that there are 71 pairs and 41 pairs of indicators with correlations reaching highly significant (*P*<0.01) and significant levels (*P*<0.05), respectively. Most correlations between indicators are statistically significant. Among the seven nutritional quality indicators of the fruit, soluble sugar content (S8) exhibits a highly significant positive correlation with the solid-acid ratio (S9), as well as a highly significant negative correlation with titratable acid (S7) and tannin content (S12). This finding indicates that higher sugar content is associated with lower levels of tannins and titratable acids.

Among the seven indicators of functional components in fruits, flavonoids (S14), polyphenols (S15), anthocyanins (S16), resveratrol (S17), chlorogenic acid (S18), and ascorbic acid content (S19) exhibit highly significant positive correlations. The synthesis pathways of these substances in plants demonstrate that resveratrol is synthesized via the flavonoid biosynthetic pathway, while flavonoids are produced through their own biosynthetic pathway, both of which utilize p-coumaroyl-CoA, synthesized via the phenylpropanoid pathway, as a common precursor. This is a key reason why resveratrol content (S17) and total flavonoid content (S14) exhibit a highly significant positive correlation. Additionally, iron-reducing ability (S22) exhibits a significant positive correlation with flavonoids (S14), polyphenols (S15), anthocyanins (S16), resveratrol (S17), and chlorogenic acid (S18).

Among the nine indicators of fruit antioxidant capacity and enzyme activity, only iron-reducing ability (S22) and LOX activity (S27) exhibit a highly significant positive correlation, while other indicators exhibit no significant correlations. Iron-reducing ability is highly significantly positively correlated with flavonoids and ascorbic acid content, which indicates a strong association among polyphenols, flavonoids, and antioxidant capacity in mulberry fruits. This suggests that polyphenols and flavonoids are important for the antioxidant properties of mulberry fruits, and that higher concentrations are associated with enhanced antioxidant capacity. The correlation analysis indicates that a degree of association and relative independence exist among the fruit quality traits of mulberry. These indicators influence the sensory quality, intrinsic quality, antioxidant capacity, and enzyme activity of mulberry fruits to varying extents.

#### 3.5.2 Principal component analysis

Principal component analysis (PCA) helps reduce dimensionality by identifying the principal components that explain the greatest variance in the data, allowing focus on the most critical variables affecting fruit quality (Sun et al., 2018). To condense the data on fruit quality traits from various mulberry varieties into a smaller set of representative variables, minimize errors, and achieve a more reasonable evaluation, principal component analysis (PCA) was employed to identify core evaluation indicators. The entropy weight method was utilized for weight assignment, while grey relational analysis was employed for comprehensive evaluation. Principal component analysis was performed on 28 indicators related to sensory quality, nutritional quality, functional components, and antioxidant properties of fruits from 21 mulberry varieties. Based on the principle of eigenvalues ≥ 1, 7 principal components were extracted (**Table 7**). The first 7 principal components accounted for 88.424% of the cumulative variance, capturing the majority of information within the overall dataset, with a total eigenvalue of 24.759, effectively representing the 28 fruit traits.

**Table 7:** Principal component eigenvalues and variance contribution rate of fruit quality of different mulberry varieties.

Component	Eigenvalues	Variance contribution rate/%	Accumulated variance contribution rate/%
1	13.363	47.726	47.726
2	2.639	9.423	57.150
3	2.352	8.401	65.550
4	2.126	7.591	73.142
5	1.776	6.343	49.485
6	1.384	4.943	84.429
7	1.119	3.996	88.424

The eigenvalue of the first principal component is 13.363, corresponding to a contribution rate of 47.726%. The second principal component has an eigenvalue of 2.639 and a contribution rate of 9.423%. The third principal component has an eigenvalue of 2.352 and a contribution rate of 8.401%. The fourth principal component has an eigenvalue of 2.126 and a contribution rate of 7.591%. The fifth principal component has an eigenvalue of 1.776 and a contribution rate of 6.343%. The sixth principal component has an eigenvalue of 1.384 and a contribution rate of 4.943%. The seventh principal component has an eigenvalue of 1.119 and a contribution rate of 3.996%. According to the results in **Table 8**, the 12 indicators—resveratrol content, polyphenol content, anthocyanin content, tannin content, soluble sugar content, chlorogenic acid content, flavonoid content, iron-reducing ability, solid-acid ratio, MDA content, ascorbic acid content, and fruit shape index—encompass the majority of traits among the 28 indicators, collectively accounting for 88.424% of the variance.

**Table 8:** Principal component factor loading matrix of fruit quality of different varieties of mulberry.

Index	F1	F2	F3	F4	F5	F6	F7
Resveratrol content	0.963	-0.031	-0.096	0.009	-0.037	-0.059	0.089
Polyphenol content	0.954	-0.181	-0.126	0.038	0.106	-0.036	0.053
Anthocyanin content	0.945	-0.211	-0.155	0.051	-0.035	0.089	0.049
Tannin content	0.933	0.142	-0.115	0.055	0.058	0.097	0.106
Soluble sugar content	-0.925	-0.113	0.048	0.127	0.132	0.016	0.090
Chlorogenic acid content	0.915	-0.068	-0.125	0.068	0.209	-0.037	-0.096
Flavonoid content	0.910	-0.214	-0.219	0.088	0.097	-0.009	-0.106
Iron reducing ability	0.892	-0.183	-0.174	0.060	0.146	-0.098	0.008
Solid acid ratio	-0.875	0.020	0.172	0.201	0.148	0.194	-0.066
MDA content	0.849	-0.308	-0.016	0.050	-0.186	0.088	-0.029
Ascorbic acid content	0.811	-0.047	-0.234	0.340	0.271	0.132	0.097
Water content	0.778	-0.009	0.423	-0.044	-0.070	-0.161	-0.140
Titratable acid content	0.773	-0.141	-0.050	-0.034	-0.185	-0.280	0.268
Carotenoid content	0.721	0.126	-0.037	-0.416	0.147	0.014	0.277
Soluble solids content	-0.673	0.014	-0.332	0.434	0.010	0.023	-0.411
Longitudinal diameter of fruit	0.644	0.240	0.490	0.478	0.036	-0.099	-0.117
Cross diameter of fruit	0.626	0.318	0.429	-0.264	0.280	0.046	-0.322
Single fruit weight	0.576	0.357	0.495	0.215	0.349	-0.150	-0.201
LOX activity	0.154	-0.783	-0.057	-0.184	-0.305	-0.054	-0.255
POD activity	0.246	0.719	-0.127	-0.181	-0.252	0.313	-0.070
Free amino acid content	0.384	0.581	0.213	-0.456	-0.375	-0.180	0.063
CAT activity	0.065	0.482	-0.702	0.162	0.125	0.116	0.165
Fruit shape index	0.271	0.041	0.205	0.836	-0.224	-0.133	0.100
Ability to scavenge ABTS free radicals	-0.217	-0.364	0.229	-0.300	0.671	0.308	0.124
PPO activity	-0.093	0.363	-0.502	0.002	0.558	-0.344	-0.114
SOD activity	0.462	-0.101	0.332	0.076	0.144	0.657	0.079
Soluble protein content	0.520	0.188	-0.24	0.108	-0.358	0.579	-0.227
Ability to scavenge DPPH free radicals	-0.371	0.107	0.273	0.335	-0.042	0.060	0.599

#### 3.5.3 Determining the weights of evaluation indicators using the entropy weight method

Based on the entropy weight method for determining factor weights, the fruit quality evaluation indicators from 21 mulberry varieties were standardized and subsequently weighted. The results, shown in **Table 9**, indicate that among the 12 indicators, anthocyanin content, MDA content, and flavonoid content have low entropy values, high variability coefficients, and large variance gradients, thereby receiving higher weights of 0.129, 0.126 and 0.111, respectively.

**Table 9:** Entropy weight method to determine the weight of 12 evaluation indicators.

Index	Entropy value	Coefficient of variance	Weights
Fruit shape index	0.899	0.101	0.056
Soluble sugar content	0.938	0.062	0.034
Solid acid ratio	0.903	0.097	0.054
Tannin content	0.850	0.150	0.083
Flavonoid content	0.799	0.201	0.111
Polyphenol content	0.840	0.160	0.088
Anthocyanin content	0.766	0.234	0.129
Resveratrol content	0.875	0.125	0.069
Chlorogenic acid content	0.823	0.177	0.097
Ascorbic acid content	0.867	0.133	0.073
Iron reducing ability	0.854	0.146	0.080
MDA content	0.770	0.230	0.126

#### 3.5.4 Grey relational analysis method

The Grey Relational Analysis (GRA) method can simultaneously evaluate multiple quality attributes. By calculating the degree of association between different variables and reference or target values, GRA allows for ranking based on their proximity to the desired quality standards (Cao et al., 2024). According to grey system theory, the maximum values of each indicator across all tested varieties were utilized as optimal values to establish the reference sequence. The 12 indicators of fruit from 21 mulberry varieties were processed to remove dimensions, and absolute differences were subsequently calculated. Due to the differing importance of the quality traits of mulberry fruit, the final relational degrees were derived based on weights established through the entropy weight method. The comprehensive evaluation results of mulberry fruit quality for different varieties were determined through the weighted grey relational degree calculation method (**Table 10**). According to **Table 10**, the fruit quality of ‘Da 10’, ‘Feng Guo Sang’, ‘He Lan Sang’, ‘Lv Shen Zi’ and ‘Ri Ben Guo Sang’ is notably high, yielding weighted relational degrees of 0.817, 0.765, 0.757, 0.739, and 0.725, respectively.

**Table 10:** Weighted correlation of fruit quality and ranking of different varieties of mulberry.

Varieties	Weighted relevance	Ranking
Da 10	0.817	1
Feng Guo Sang	0.765	2
He Lan Sang	0.757	3
Lv Shen Zi	0.739	4
Ri Ben Guo Sang	0.725	5
He Zhen Zhu	0.657	6
Ju Shen	0.634	7
Tang 10	0.608	8
Hong Guo 1	0.608	9
Lv Shen Zi 1	0.605	10
Da Bai E	0.467	11
Ji Gui Hua	0.445	12
Gui Hua Mi	0.445	13
Xiao Bai E	0.443	14
Da Yi Bai	0.427	15
Da Ma Ya	0.425	16
Jiang Mi Guo Sang	0.424	17
Lv Shen Zi 2	0.422	18
Tian Sang 202	0.420	19
Bai Shen 2	0.420	20
Su Bai Shen	0.406	21

## 4 Discussions

### 4.1 Quality of fruits from different mulberry varieties

Mulberry trees have long been valued for their multifunctionality, and the quality of their fruit varies significantly among different varieties due to genetic factors, environmental conditions, and cultivation practices. The comprehensive evaluation of mulberry fruit quality typically encompasses a range of factors, including appearance, nutritional content, and functional components. The appearance quality of the fruit is a key determinant of its value and market competitiveness, with color and shape playing a direct role in consumer purchasing decisions. The size of the fruit plays a crucial role in consumer preferences, especially for fresh fruit, with larger, more robust fruits generally regarded as more appealing (Li et al., 2024b). In this study, a comprehensive analysis was performed on various indicators, including fruit shape index, individual fruit weight, and water content. Varieties such as ‘Ju Shen’, ‘Lv Shen Zi’, and ‘He Lan Sang’ exhibited notable advantages in appearance quality. Although the fruit shape index of ‘Da 10’ was significantly higher than those of other varieties, its individual fruit weight and water content were comparatively unremarkable. All three of these varieties exhibit a purple-black or purple-red coloration. Previous studies have demonstrated that varieties with darker pigmentation, particularly black mulberries, are highly valued due to their rich color, which is indicative of a high anthocyanin content, a known antioxidant. Moreover, color is linked to flavor intensity, with darker mulberries generally exhibiting a stronger sweetness and acidity (Adarsh et al., 2024).

In fruit production and promotion, nutritional value, alongside yield, is a key factor in evaluating fruit quality. This study identified varieties such as ‘Da Bai E’, ‘Da Yi Bai’, ‘Ji Gui Hua’, ‘Xiao Bai E’, ‘Jiang Mi Guo Sang’, and ‘Gui Hua Mi’ as particularly outstanding in terms of solid-acid ratio. Previous studies have demonstrated that higher TSS levels correlate with increased sweetness, making the fruit more suitable for fresh consumption (Jiao et al., 2023). Free amino acids in fruits supplement the human body with essential amino acids, while soluble proteins contain essential peptides and other bioactive components (Shih et al., 2010), contributing to health benefits. This study found no significant variation in soluble protein content across the varieties. However, when both free amino acids and soluble proteins were considered, varieties such as ‘Hong Guo 1’, ‘Tang 10’, and ‘Su Bai Shen’ emerged as prominent, enhancing their potential as functional foods.

As health awareness grows, plant-derived functional components, particularly phenolic compounds such as flavonoids and anthocyanins, have garnered significant attention (Hu et al., 2019). In this study, the ranking of functional components, including carotenoids, flavonoids, polyphenols, anthocyanins, and resveratrol, revealed that varieties such as ‘Da 10’, ‘He Lan Sang’, ‘Lv Shen Zi’, and ‘Ju Shen’ were particularly prominent. These compounds are recognized for their anti-inflammatory, anticancer, and anti-aging properties. Among them, the anthocyanin content in ‘Ri Ben Guo Sang’ was comparatively low, whereas the levels of other components, particularly polyphenols, were relatively high, with the polyphenol content measuring 148.37 mg/g. These compounds significantly influence fruit hardness, color, flavor, and storage characteristics, making their content a critical indicator of nutritional quality (Marta et al., 2020; Onwuchekwa et al., 2014; Maja et al., 2020). Black mulberries are known to have the highest anthocyanin content, which contributes to their deep color and health-promoting properties (Sun et al., 2018). In this study, ‘Feng Guo Sang’ demonstrated particularly high levels of polyphenols, anthocyanins, and flavonoids, whereas the content of other functional components remained at moderate levels.

Numerous studies have shown that fruits contain various antioxidant compounds that neutralize free radicals, mitigate aging, and enhance immune function (Patricia and Syaputri, 2021). In this study, a comprehensive analysis was conducted on DPPH radical scavenging ability, ATBS radical scavenging ability, iron-reducing capacity, and MDA content. ‘Da Bai E’ demonstrated strong antioxidant activity, whereas the DPPH radical scavenging ability of ‘Da Yi Bai’ and ‘Su Bai Shen’ was comparatively weaker. Beyond anthocyanins, mulberries also contain a variety of other compounds that contribute to enhanced antioxidant activity. Varieties such as Black Mulberry and White Mulberry have been shown to possess significant antioxidant potential, increasing their appeal to health-conscious consumers (Adarsh et al., 2024).

Enzyme activity in fruit is crucial for post-harvest quality, the ripening process, and nutritional value. Specific enzymes influence the texture, flavor, color, and nutritional characteristics of fruit. Higher PPO activity is typically linked to increased browning, which is undesirable in fresh fruits but beneficial in processing. During processing, browning may need to be controlled to preserve the color (Wang et al., 2017). In this study, the PPO activity of varieties such as ‘Lv Shen Zi 2’, ‘Ri Ben Guo Sang’, ‘He Lan Sang’, ‘Lv Shen Zi 1’, and ‘Da Ma Ya’ was significantly high, offering a distinct advantage for subsequent processing and utilization. ‘Jiang Mi Guo Sang’ demonstrated the highest SOD activity, ‘Hong Guo 1’ exhibited the highest POD activity, and ‘Lv Shen Zi 1’ displayed the highest CAT activity. Considering the combined activity of the three antioxidant enzymes, varieties such as ‘Da 10’, ‘Hong Guo 1’, and ‘Jiang Mi Guo Sang’ exhibited strong overall antioxidant enzyme activity. Antioxidant enzymes, including SOD, CAT, and POD, are crucial for protecting fruit tissue from oxidative damage during ripening and post-harvest storage. These enzymes scavenge reactive oxygen species (ROS) and are linked to the maintenance of fruit quality and shelf life. The ethanol extract of mulberry jelly can protect neuronal cells from oxidative stress-induced apoptosis by enhancing the production of antioxidant enzymes and the formation of brain-derived neurotrophic factor (Bisma et al., 2021).

The comprehensive evaluation of mulberry fruit quality involves a multifaceted analysis of appearance, nutritional quality, functional properties, as well as post-harvest shelf life and tree productivity. Different varieties offer distinct advantages depending on their intended use. To meet the increasing demand for mulberry fruit in both commercial markets and health-oriented product lines, further research into breeding techniques and cultivation methods is essential.

### 4.2 Comprehensive evaluation of different mulberry fruit varieties

The sensory quality, nutritional value, functional components, and antioxidant properties of fruit are critical factors influencing consumer selection. As a result, fruit quality has long been a primary focus for breeders, and the development of scientifically robust methods for evaluating fruit quality is essential for the selection of superior cultivars. To conduct a comprehensive evaluation of mulberry fruit quality across different varieties, sophisticated multivariate techniques are required to manage the complexity and diversity of the variables involved. This study utilizes Principal Component Analysis (PCA) and Grey Relational Degree (GRD), both of which are powerful tools for assessing overall fruit quality and identifying key factors that influence it. These methods are particularly effective for managing the variability of quality parameters and distilling complex datasets into actionable insights.

In this study, principal component analysis (PCA) was employed to simplify 28 quality indicators, leading to the extraction of seven principal components, with a cumulative contribution rate of 88.424%. Twelve core indicators were selected, including resveratrol, polyphenol, anthocyanin, tannin, soluble solids, chlorogenic acid, flavonoid, iron-reducing capacity, solid-acid ratio, MDA, ascorbic acid, and fruit shape index. The weight of each indicator was determined using the entropy weight method, followed by a comprehensive evaluation using the grey relational analysis method. Correlation analysis revealed that, among the 12 core indicators, all except the fruit shape index were significantly correlated. Significant or highly significant correlations were also found with water content, titratable acid content, and carotenoid content, suggesting that moisture and acidity in mulberries may influence the levels of bioactive compounds. With the exception of ascorbic acid content, the remaining 10 indicators exhibited significant or highly significant correlations with soluble solids content, suggesting that soluble solids content in mulberries does not significantly influence vitamin C levels. Among these, soluble sugars, tannins, anthocyanins, resveratrol, ascorbic acid, and MDA were significantly or highly significantly correlated with soluble protein content, suggesting that these components in mulberries are interrelated.

The quality characteristics of mulberry fruit vary significantly across different geographic regions. Principal Component Analysis (PCA) identified two principal components (PC1 and PC2) that are strongly associated with specific bioactive compounds and antioxidant properties. PC1 is closely correlated with MAC, FRAP, and C3G, indicating a robust relationship with antioxidant activity and its key constituents. Meanwhile, PC2 is associated with titratable acidity (TA), DPPH radical scavenging, and OH radical scavenging, although its correlation with C3G is weaker. The significant correlation between Total Phenolic Content (TPC) and DPPH suggests that TPC plays a crucial role in DPPH radical scavenging. This study reveals that geographic region significantly impacts the content of bioactive compounds in mulberry fruit, thereby affecting its antioxidant capacity (Chen et al., 2022). Sun et al. (Sun et al., 2018) used PCA to evaluate the quality of mulberry fruits from different varieties, and the analysis showed that the three principal components explained 80.60% of the total variance of fruit quality data, indicating that the contents of rutin, anthocyanin and vitamin C were positively correlated with titrable acid. Titrable acids play a key role in the stability of anthocyanins, vitamin C and rutin. Li et al. (Li et al., 2024c) conducted a similar PCA analysis to assess grape fruit quality, identifying critical indicators such as fruit weight, dimensions, reducing sugar content, soluble solids, flavonoids, anthocyanins, and tannins.

Principal Component Analysis (PCA) and Grey Relational Degree (GCD) are both effective methods for evaluating fruit quality, and their combination provides a more comprehensive analysis. PCA reduces the dimensionality of the data and identifies the key factors influencing quality, while GCD ranks varieties based on their proximity to ideal quality standards. This study applied both methods to conduct a thorough evaluation of mulberry fruits from different varieties, offering a detailed understanding of their quality. The findings provide scientific support for the selection and breeding of mulberry varieties in Hebei Province, contributing to variety selection, breeding programs, and quality control in commercial production. However, this paper focuses only on the fruit quality of mulberry varieties, and does not address the potential applications of the tree’s leaves, buds, and other parts. Future research will aim to further evaluate these aspects, completing the development of a comprehensive and rigorous scientific evaluation system.

## 5 Conclusions

To comprehensively evaluate the main mulberry varieties planted in Hebei Province, this study selected 21 mulberry varieties and conducted an in-depth assessment using Principal Component Analysis (PCA) and Grey Relational Degree (GCD). The evaluation covered key factors including appearance quality, nutritional quality, functional components, antioxidant capacity, and enzyme activity. Through PCA, 12 core indicators were identified, and their weights were determined using the entropy weighting method. These indicators were then dimensionless processed based on grey system theory, and correlation coefficients were calculated to assess each variety. The top six varieties, based on their comprehensive scores, were: ‘Da 10’, ‘Feng Guo Sang’, ‘He Lan Sang’, ‘Lv Shen Zi’, ‘Ri Ben Guo Sang’, and ‘He Zhen Zhu’, with corresponding weighted relational degrees of 0.817, 0.765, 0.757, 0.739, 0.725, and 0.657, respectively.

In conclusion, the ‘Da 10’ variety features medium-sized, elongated cylindrical fruits with a purple-black coloration. It has a composition that includes 11.50% soluble sugar, 1.91% titratable acid, a solid-acid ratio of 6.02, 194.67 mg/g free amino acids, 21.91% soluble solids, 12.72 µg/g soluble proteins, 2.85 mg/g tannins, 96.45 mg/g flavonoids, 145.66 mg/g polyphenols, 2.57 mg/g resveratrol, 34.71 mg/g chlorogenic acid, 760.63 mg/100g ascorbic acid, and 60.62 mg/g anthocyanins. Overall, it is characterized by high quality and exceptional flavor. These attributes provide a strong theoretical basis for the selection, breeding, and promotion of mulberry varieties in Hebei Province. Additionally, significant differences in fruit quality traits were observed across the 21 mulberry varieties. Varieties with outstanding appearance quality are suitable for fresh consumption, while those with superior nutritional content can be used for products such as mulberry wine. Varieties rich in functional components may have medicinal applications, thereby expanding possibilities for future processing and utilization.

## Data Availability

The raw data supporting the conclusions of this article will be made available by the authors, without undue reservation.
